# An Expanded Narrative Review of Neurotransmitters on Alzheimer’s Disease: The Role of Therapeutic Interventions on Neurotransmission

**DOI:** 10.1007/s12035-024-04333-y

**Published:** 2024-07-16

**Authors:** Enes Akyuz, Alina Arulsamy, Feyza Sule Aslan, Bugra Sarisözen, Beyzanur Guney, Abdulhekim Hekimoglu, Beyza Nur Yilmaz, Thaarvena Retinasamy, Mohd. Farooq Shaikh

**Affiliations:** 1 Department of Biophysics, International School of Medicine, University of Health Sciences, Istanbul, Turkey; 2 Neuropharmacology Research Laboratory, Jeffrey Cheah School of Medicine and Health Sciences, Monash University Malaysia, 47500 Bandar Sunway, Selangor Malaysia; 3 School of Medicine, Marmara University, Istanbul, Turkey; 4 School of Medicine, Tekirdağ Namık Kemal University, Tekirdağ, Turkey; 5 International School of Medicine, University of Health Sciences, Istanbul, Turkey; 6 School of Dentistry and Medical Sciences, Charles Sturt University, Orange, New South Wales 2800 Australia; 7 Department of Pediatrics, School of Medicine and Public Health, University of Wisconsin-Madison, Madison, WI USA

**Keywords:** Alzheimer’s disease, Neurotransmitters, Glutamate, Acetylcholine, Amyloid-β, Neurodegeneration

## Abstract

Alzheimer’s disease (AD) is a progressive neurodegenerative disease. The accumulation of amyloid-β (Aβ) plaques and tau neurofibrillary tangles are the key players responsible for the pathogenesis of the disease. The accumulation of Aβ plaques and tau affect the balance in chemical neurotransmitters in the brain. Thus, the current review examined the role of neurotransmitters in the pathogenesis of Alzheimer’s disease and discusses the alterations in the neurochemical activity and cross talk with their receptors and transporters. In the presence of Aβ plaques and neurofibrillary tangles, changes may occur in the expression of neuronal receptors which in turn triggers excessive release of glutamate into the synaptic cleft contributing to cell death and neuronal damage. The GABAergic system may also be affected by AD pathology in a similar way. In addition, decreased receptors in the cholinergic system and dysfunction in the dopamine neurotransmission of AD pathology may also contribute to the damage to cognitive function. Moreover, the presence of deficiencies in noradrenergic neurons within the locus coeruleus in AD suggests that noradrenergic stimulation could be useful in addressing its pathophysiology. The regulation of melatonin, known for its effectiveness in enhancing cognitive function and preventing Aβ accumulation, along with the involvement of the serotonergic system and histaminergic system in cognition and memory, becomes remarkable for promoting neurotransmission in AD. Additionally, nitric oxide and adenosine-based therapeutic approaches play a protective role in AD by preventing neuroinflammation. Overall, neurotransmitter-based therapeutic strategies emerge as pivotal for addressing neurotransmitter homeostasis and neurotransmission in the context of AD. This review discussed the potential for neurotransmitter-based drugs to be effective in slowing and correcting the neurodegenerative processes in AD by targeting the neurochemical imbalance in the brain. Therefore, neurotransmitter-based drugs could serve as a future therapeutic strategy to tackle AD.

## Introduction

Alzheimer’s disease (AD) is a neurodegenerative disease and the most common form of dementia, characterized by the progressive deterioration of memory and cognitive abilities. AD, which once had mainly hereditary origins such as its association with apolipoprotein E (APOE) alleles, now has various precursor risk factors such as traumatic brain injury, epilepsy, and stroke [1–4]. At the molecular level, extracellular amyloid β (Aβ) plaques and intracellular tau-containing neurofibrillary tangle (NFT) accumulation are the main drivers of AD progression. This accumulation leads to oxidative stress and inflammatory reactions, thereby contributing to synaptic dysfunction and neuronal degeneration, all of which compromise the cognitive functionality in people with AD [5, 6].

Aβ, in its miniscule physiological concentrations of nano molars and picomolars, is a monomeric protein important for proper functioning of synaptic transmission [7]. In pathologies such as AD, Aβ concentration is severely dysregulated resulting in Aβ monomers to progressively aggregate into soluble Aβ oligomers or non-soluble Aβ fibrils [8]. While the Aβ fibrils are responsible for Aβ plaque lesions seen in the AD patients, water-soluble Aβ oligomers are able to accumulate through the brain which is the prominent reason for the cognitive damage seen in AD [9]. The accumulation of Aβ oligomers results in impairing the neurochemical functionality especially the neurotransmitter release [10, 11]. Neurotransmitters are essential chemicals responsible for communication between neurons. They exert their associated effects through their specific receptors. Neurotransmitter transporters, on the other hand, regulate the concentration of neurotransmitters in the synaptic gap and ensure the healthy and harmonious execution of receptor activation. Together with their receptors and transporters, neurotransmitters form the basis of interneuronal (synaptic) transmission. In the presence of Aβ plaques and tau NFTs, the level of various neurotransmitters was imbalanced [12] and so were the receptor localization and expression, which have been reported to be impaired [13, 14]. In addition, functional factors like altered electrical currents associated with these receptors were also previously reported [15].

Disruption of cholinergic neurotransmission exacerbates Aβ-related cognitive impairment evident in a preclinical AD model [16]. Similarly, dysfunction of the serotonergic system was found in the hippocampus of post-mortem brain samples of individuals with AD patients. In addition, impaired norepinephrine and dopaminergic neurotransmitter systems were also observed in the dorsolateral and anterior prefrontal cortex of AD-affected brains [17]. Therefore, to restore neurotransmitter balance in AD and prevent the progression of the disease and its associated cognitive impairments, the contribution or role of neurotransmitters in the pathophysiological mechanism of AD needs to be elucidated. In this narrative review, the role of various key neurotransmitters in AD pathology, including the function of their respective receptors and transporters involved in the neuro-communication pathway, has been discussed, in hopes to illuminate a potential therapeutic target/strategy against AD progression.

## Amino Acid–based Neurotransmitters

### Glutamate

Glutamate is one of the main excitatory neurotransmitters in the central nervous system (CNS). Glutamate receptors can be divided into two types: metabotropic glutamate receptors (mGluR) and ionotropic glutamate receptors (iGluR). The widespread expression and therapeutic utility of mGluR ligands in the CNS entail it as a suitable drug target for glutamate-associated neurological disorders such as AD [18–20]. On the other hand, the iGluR family comprises of the α-amino-3-hydroxy-5-methyl-4-isoxazolepropionic acid receptors (AMPAR), N-methyl-d-aspartate receptors (NMDAR), and kainate receptors (KAR) which act as cation channels in the CNS [21]. NMDARs, which are crucial in memory and learning, may interact with Aβ and cause excitotoxicity. Extrasynaptic NMDARs cause cell death and neuronal damage in the neurodegeneration mechanism in AD while synaptic NMDARs ensure cell survival [22, 23]. Systemic inflammation may also alter protein expression of iGluR subunit 1 (GluN1) in the cerebral cortex contributing to the development of AD [24].

Inflammation plays a crucial role in the context of glutamate neurotransmission, interacting with neurons, astrocytes, and microglia. The accumulation of glutamate in the environment, resulting from excitotoxicity, prompts its uptake into astrocytes through EAAT1/2. Once inside astrocytes, glutamate transforms into glutamine, aiding in the removal of synaptically released glutamate from the surroundings. This process is vital for preserving plasticity and inhibiting excitotoxicity, thereby preventing the pathogenesis of AD [25–28]. Conversely, an excess of glutamate in the environment amplifies glutamate release by activating AMPA, NMDA, and mGlu receptors in neurons and microglia. The elevated glutamate release from neurons and microglia, coupled with impaired clearance by astrocytes, leads to increased inflammation. This inflammatory response contributes to synaptic dysfunction and damages cognitive function in AD [29–31]. In a study, an inflammation model was established in astrocyte, microglia, and neuron cultures. The elevated glutamate levels during inflammation increased l-glutamate release in microglia. Additionally, heightened glutamate levels in astrocytes resulted in the downregulation of astrocyte transporters. Neuroinflammation-induced disruptions in microglia and astrocytes contribute to increased glutamate levels in the astrocyte-microglia-neuron area [32]. The regulation of astrocyte-neuron interactions may hold promise for managing cognitive function in AD by reducing Aβ accumulation and tau hyperphosphorylation [33]. Approaches targeting the modulation of astrocyte and microglia mechanisms could be a potential avenue to halt the progression of AD [34].

**Fig. 1:**
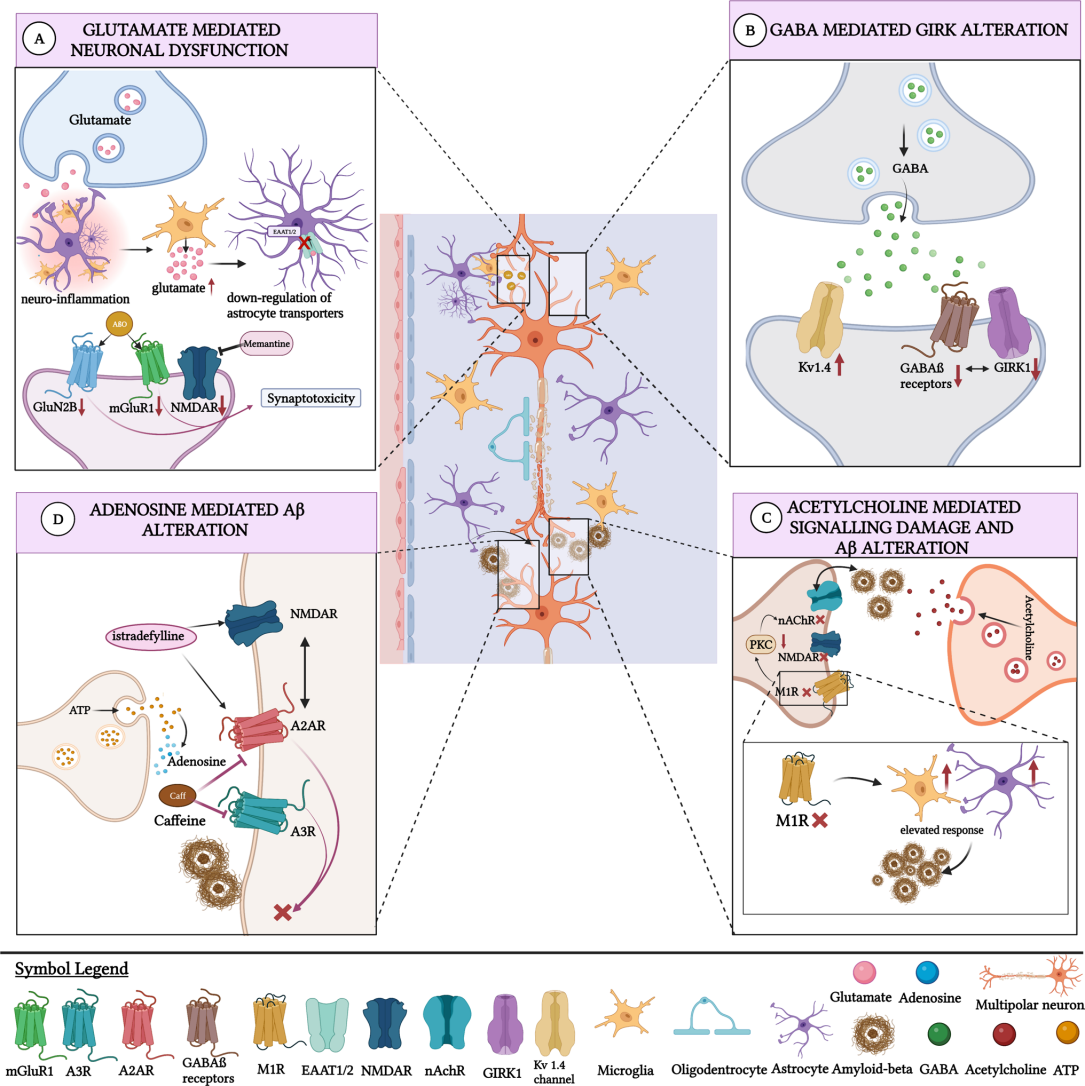
**A** The role of glutamate in AD. During inflammation, elevated glutamate levels increases glutamate release in microglia. This shows that neuroinflammation can contribute to increased glutamate levels in the astrocyte-microglia-neuron area. This heightened glutamate levels can result in the downregulation of astrocyte transporters. Memantine reduces NMDAR activity by acting as an antagonist. AβO can induce pathological mechanisms by interacting with GluN2B and MgluR1 in AD patients. This interaction causes synaptotoxicity. **B** The role of GABA in AD. GIRK1 and GABAβ receptor co-clusters appear to be decreased in AD patients. AD pathology alters GABAergic systems both functionally and structurally. APP increases the level of KV1.4 channel in GABAergic neurons. **C** The role of acetylcholine in AD. Deletion or the disturbances of M1R can lead to elevated microglial and astrocytic responses which is associated with Aß plaques. Disturbances in the acetylcholine receptor M1R are effective in PKC signal loss. These dysfunctions are effective in AD through PKC signal disturbances and changes in NMDAR expression. High Aβ density negatively affects nAchR function. Decreased nAchR has been observed in AD patients. **D** The role of adenosine in AD. ATP released from synapses is converted into adenosine and given to the synaptic gap. Caffeine, a selective antagonist, suppresses A3R and A2AR functions. Inhibition of A2AR and A3R reduces Aβ formation. A2AR antagonists such as istradefylline affect NMDAR functions. NMDA and A2AR can interact to form complexes

Besides that, NMDARs containing mGluR1 and GluN2B may have crucial roles in AD pathology (Fig. 1A). Interactions with the GluN2B subunit and mGluR1 receptor Aβ oligomers (AβO) located at the NMDA receptor in the primary cortical area reported that extracellular AβOs (2.5 μM) specifically bind together to induce pathological responses and cause synaptic disruptions. This special binding could be explained by the colocalization of mGluR1 and Aβ and the presence of Aβ and GluN2B immunoreactivities in neurites [35, 36] In AD, AβO prevents physiological activation of the cellular prion protein-mGluR5 (PrPC-mGluR5) complex by glutamate. In neurodegeneration and dementia, PrPC-mGluR5-dependent events are triggered by AβO in APP/PS1 mice [37]. Thus, the PrPC-mGluR5 complex may be targeted to correct the aberrant AβO pathway by glutamate signaling. Another study investigated the role of mGluR5 on glutamate signaling and phenotype in a mouse model of AD. The role of mGluR5 in AβO-dependent AD phenotypes was found to be different from its role in glutamate signaling [38]. Thus, mGluR5 may also serve as a new target for correction of the pathological mechanism in AD.

Despite these evidences, in a study using positron emission tomography (PET), unchanged type 1 mGluR, particularly mGluR1, was visualized in AD patients suggesting that the presence of mGluR did not change in the early stages of AD but may change in the later stages of AD [39]. Whether this time-dependent change expands to other mGluR and iGluR remains to be determined.

In addition to the dysfunction that occurs in the iGluR and mGluR families, excessive glutamate release in glial cells may also cause synaptic dysfunction by increasing excitation [30, 40]. The extracellular glutamate ratio in the brain is primarily controlled by excitatory amino acid transporters and vesicular glutamate transporters [41]. Excitatory amino acid transporters 1 and 2 (EAAT 1 AND EAAT 2) are astrocytic glutamate transporters. These transporters keep the extracellular glutamate level in balance by the transport of one H^+^ and one K^+^ versus three Na^+^ ions. Impaired EAAT expression leads to disruption of synaptic transmission, extracellular glutamate accumulation, and excitotoxicity, leading to neurodegenerative diseases such as AD [42, 43]. Since synapse loss is associated with glia glutamate transporter-1 expression and glutamate balance, the drug ceftriaxone (Cef) was investigated. Accordingly, Cef (200 mg/kg) corrected dendritic degeneration and hippocampal synapse loss via GLT-1 [44]. Microglia/macrophage activation by Cef may contribute to synaptic improvement in APP/PS1 mice. Glutamate balance in the synaptic cleft was examined via GLT-1 and slower clearance of glutamate was found in astrocytic GLT-1 presynapses with CA-3 and CA-1 neurons in C57BL/6NCrl mice [45]. The fact that GLT-1 dysfunction makes presynapses more vulnerable may introduce the concept of presynaptic disorders in brain diseases. Synaptic loss and extrasynaptic NMDA receptor activation induces the release of Aβ-triggered microglia glutamate as well. Alpha-7 nicotinic receptors caused synaptic damage by stimulating excessive glutamate secretion [46]. Normal synaptic transmission and neurobehavioral recovery can be achieved with NMDAR antagonists. The NMDA receptor antagonist memantine is frequently used in AD as a treatment drug as it suppresses the proliferation of microglial cells and stops neuronal death. A rodent preclinical AD study on primary microglia cell of C57BL/6 J mice showed that memantine (5 µM) indirectly regulated the phagocytic activity in microglia and mediated neuroinflammation [47] (Fig. 1A). The association of Aβ-related (Aβ_1–42_ peptide; 500 nM) oligomer neuronal hyperactivity with NMDAR was investigated by whole-cell patch-clamp. GLT disruption in CA1 hippocampal neurons increased NMDAR activity. The glutamate accumulation that occurred in this situation strengthened the synaptic inputs in neurons [48]. Enhancement of synaptic inputs and increased cell excitability may play a role in neuronal hyperactivity initiated by impaired glutamate reuptake. In addition, astrocytic glutamate release can be activated by extrasynaptic NMDARs (eNMDAR). Miniature excitatory postsynaptic currents (mEPSC) are seen after increased eNMDAR activity [46]. In particular, drugs such as NitroMemantine that inhibit extrasynaptic NMDAR can protect synapses from Aβ.

Similarly, another treatment drug for AD which act as a glutamate modulator, Riluzole (50 mg twice a day), preserved the cerebral glucose level and cognitive performance after administration [49]. The accumulation of Aβ also brings about electrophysiological disorders in AD. Aβ, which may be responsible for pore formation in neuronal membranes, causes an increase in intracellular calcium, ethidium bromide flux, and membrane conductivity [50].

Interestingly, genome-wide association studies (GWAS) can not only detect the locations of genes that increases the risk towards AD, such as APOE4, but can also determine the genetic variations in glutamate signaling [51]. The level of glutamate signaling protein in the prefrontal cortex in AD and its relationship with the APOE4 genotype may determine the expression levels of glutamate receptors and the amount of synaptic protein in the prefrontal cortex [52], thus indicating that targeting effort of glutamate receptors in AD may be dependent on the APOE4 genotype (Table 1). In addition, changes in glutaminergic signaling may also occur as Aβ plaques develop. When glutamate levels were measured by microelectric array in a study involving double transgenic mice expressing the presenilin 1 (PS1-dE9) genes (AβPP/PS1), the Aβ plaque deposition was temporarily but anatomically aligned with high glutamate levels in the brain [53]. Studies on AppNL-F and AppNL-G-F knock-in mice, which are a recent model of AD, are increasing. Accordingly, the level of glutamate with CA1 hippocampal neurons was investigated using Aβ to understand synaptic function, plasticity, and genetic microglial changes. As a result, it was observed that the possibility of glutamate release increased in transgenic mouse models [54]. This may be related to the acute effect of soluble Aβ plaques. This evidence suggests that accurate detection of abnormalities in genes related to glutamate synthesis and degradation enzymes, as well as changes in glutamate levels, may act as biomarkers to enable time-specific interventions for AD.

**Table 1:** The role of glutamate in AD based on previous literature

Study type	AD model	Drug and dose	Observation	Remarks	References
In vitro	Primary microglia cell of C57BL/6 J mice	Memantine (5 µM)	Memantine indirectly regulated phagocytic activity in microglia	The effect of memantine on the brain was evaluated through microglia-mediated neuroinflammation in AD	[47]
	Primary neuronal cultures of Wistar rat	–	Interaction with the GluN2B subunit and mGluR1 receptor AβO located at the NMDA receptor in the primary cortical area reported that extracellular AβOs specifically bind to induce pathological responses and cause synaptic disruptions	NMDA receptors containing mGluR1 and GluN2B may have crucial roles in AD pathology	[35]
	APdE9 mice	–	Systematic inflammation triggered changes in GluN1 and contributed to the development of AD	Quantitative information in expression may provide more accurate information for drug development for humans from animal models of AD	[24]
	Mixed culture composed of astrocytes, microglia, and neurons	–	Activation of microglia and astrocytes causes an increase in extracellular l-glutamate in the early stages of neuroinflammation	The regulation of astrocyte-neuron interactions may hold promise for managing of AD	[32]
	CA1 pyramidal neurons of male Sprague–Dawley rats	Aβ_1–42_ peptide oligomer (diluted, final concentration of 500 nM)	The glutamate accumulation strengthened the synaptic inputs in neurons	Enhancement of synaptic inputs and increased cell excitability may play a role in neuronal hyperactivity initiated by impaired glutamate reuptake	[48]
	Mixed neuronal/glial rat cerebrocortical cultures and purified rat, mouse, and human astrocytes	–	mEPSC are seen after increased eNMDAR activity	NitroMemantine that inhibit extrasynaptic NMDAR can protect synapses from Aβ	[46]
	Hippocampal neurons of (C57BL/J6) or Sprague–Dawley rat embryos	–	Aβ may be responsible for pore formation in neuronal membranes	This causes an increase in intracellular calcium, ethidium bromide flux, and membrane conductivity	[50]
In vitro, in vivo	APP/PS1 mice	–	The role of mGluR5 in AβO-dependent AD phenotypes was found to be different from its role in glutamate signaling	mGluR5 may be a new target for correction of the pathological mechanism in AD	[38]
	Transgenic mice model, purified rat, mouse, and human astrocytes	NitroMemantin (5–10 µM)	α7 nicotinic receptors caused synaptic damage by stimulating excessive glutamate secretion	Normal synaptic transmission and neurobehavioral recovery may be achieved with NMDAR antagonists including NitroMemantine	[46]
	App^NL−F^ and App^NL−G−F^ knock-in mice	–	The level of glutamate with CA1 hippocampal neurons was investigated using Aβ to understand synaptic function, plasticity, and genetic microglial changes	It was observed that the possibility of glutamate release increased in transgenic mouse models	[54]
	APP/PS1 mice, neurons (PC12 cells), and astrocytes (C6 cells)	Sodium acetate (67.5 mM), sodium butyrate (40 mM), sodium propionate (25 mM)	Regulation of astrocyte-neuron interaction contributes to the regulation of cognitive function in AD by reducing Aβ accumulation and tau hyperphosphorylation	Activating astrocyte-neuron glutamate transport pathways may be a new approach for the treatment of AD	[33]
In vivo	C57BL/6 J and AβPP/PS1 mice	–	Glutamate levels were measured by microelectric array in double transgenic mice expressing the PS1-dE9 genes (AβPP/PS1). Plaque deposition was temporarily anatomically aligned with high glutamate levels	Hippocampal glutamate changes observed for time-specific interventions for AD may serve as biomarkers	[53]
	APP/PS1 mice (*n* = 9)	–	In neurodegeneration and dementia, PrPC-mGluR5-dependent events were triggered by AβO	The PrPC-mGluR5 complex may be targeted to correct the aberrant AβO pathway by glutamate signaling	[37]
	APP/PS1 mice	Ceftriaxone (200 mg/kg)	Cef corrected dendritic degeneration and hippocampal synapse loss via GLT-1	Microglia/macrophage activation by Cef may contribute to synaptic improvement	[44]
	C57BL/6NCrl mice	–	Slower clearance of glutamate was found in astrocytic GLT-1 presynapses with CA-3 and CA-1 neurons	GLT-1 dysfunction makes presynapses more vulnerable may introduce the concept of presynaptic disorders in brain diseases	[45]
Human study	AD patients (*n* = 94)	Riluzole (50 mg twice a day)	Structural and molecular changes of Riluzole as another glutamate modulator revealed that cerebral glucose level and cognitive performance were preserved in AD	Riluzole may be accepted as a potential agent	[49]
	829 AD patients (*n* = 829), HC (*n* = 535)	–	Glutamate signaling in AD determined that genetic variation in glutamate signaling contributes to the genetic risk of AD	GWAS may be recommended for the detection of functional biological networks to treat and prevent this neurodegenerative disease	[51]
	AD patients (*n* = 10)	–	The presence of mGluR1 did not change in the early stages of AD	However, the presence of mGluR1 may change in the later stages of AD	[39]
	AD patients (*n* = 59), HC (*n* = 10)	–	The level of glutamate signaling protein in the prefrontal cortex in AD and its relationship with the APOE*4 genotype determined that the expression of glutamate receptors decreased and the amount of synaptic protein in the prefrontal cortex changed	The targeting effort of glutamate receptors in AD may be dependent on the APOE*4 genotype	[52]

*AD* Alzheimer’s disease, *AβO* amyloid-β oligomer, *HC* healthy controls, *GluN1* N-methyl-d-aspartate receptor-1, *GWAS* genome-wide association studies, *mGluR5* metabotropic glutamate receptor-5, *mEPSC* miniature excitatory postsynaptic currents, *NMDAR* N-methyl-d-aspartate receptor, *PET* positron emission tomography

In AD, disruptions to the transmission of the glutamate can be attributed to oxidative stress and nitrosative stress. Oxidative and nitrosative stress result from an imbalance in redox reactions, leading to the excessive generation of free radicals. The presence of reactive oxygen species (ROS) due to oxidative stress negatively impacts synaptic plasticity, contributing to cognitive decline [55–57]. ROS also induces excitotoxic effects by increasing calcium influx through glutamate receptors, particularly the NMDA receptors. The interaction between NMDA receptors and ROS further contributes to memory loss by reducing receptor expression and causing synapse dysfunction through increased tau hyperphosphorylation [58, 59]. A study on Alzheimer’s patients revealed that oxidative stress disrupts glutamate intake, potentially upsetting the glutamate balance and leading to memory loss [60]. Nitrosative stress, characterized by the emergence of reactive nitrogen species (RNS), is another crucial factor in AD’s pathogenesis. This stress occurs during the production of nitric oxide from arginine via nitric oxide synthase (NOS), with NOS activity in neuron cells influencing glutamatergic neurotransmission. Excessive nitrosative stress may disrupt glutamate neurotransmission, contributing to impaired function in AD [61]. Addressing free radicals, including ROS and RNS, could be a potential strategy to alleviate cognitive dysfunction in AD [62].

### GABA

Gamma-aminobutyric acid (GABA) is the primary inhibitory neurotransmitter in the CNS. GABA is involved in triggering fast and slow synaptic inhibition and suppressing excitation of neuronal activity through its receptors and effectors. In terms of cognition, GABA plays a role in visual orientation, active memory, and mechanisms related to chronic pain [63–65].

GABA has two distinct receptors. GABA_A_ receptors function as major inhibitory receptors of the brain. These receptors act as chlorine-selective anion channels responsible for rapid synaptic inhibition. GABA receptors are usually pentameric proteins containing different subunits. Six subunits (α, β, γ, ρ, ε, δ, θ, π) have been defined for GABA_A_ [66, 67]. Activation of post-synaptic and presynaptic GABA_A_ receptors causes phasic inhibition defined by short-acting fast-acting currents. High-affinity GABA_A_ receptors in the extra-synaptic region are responsible for tonic inhibition [68, 69].

Structurally similar to GABA_A_ receptors, GABAC receptors are chlorine-selective anion channels similar with GABA_A_ receptors [70]. Similarities between two receptors and the fact that GABAc receptors are much more simpler in the context of subunit composition resulted in a postulation that GABAc receptors should be considered a subtype of GABAa receptors (GABA_A-p_) [70, 71]. With different electromechanical and pharmacological characteristics, GABAc receptors are primarily expressed in the retina and regulates timing and duration of inhibitory currents taking part in the visual processing [71–73]. Finally GABAc receptors, despite being similar to GABAA receptors, are yet to be investigated as a standalone AD pathogenetic factor.

Apart from rapid synaptic inhibition, the GABAergic system can also create slow and long-term inhibition over its other receptor, GABA_B_. Presynaptic GABA_B_ receptors inhibit the release of GABA and other neurotransmitters into the synaptic space by reducing calcium entry into the neuron. Postsynaptic GABA_B_ receptors, on the other hand, are responsible for hyperpolarizing the cell by increasing efflux of potassium ions [74, 75].

The proper functioning of GABA receptors depends on proper regulation of the GABA concentration in the intercellular space. GABA concentration in the intercellular space is regulated by GABA transporters (GAT) [76]. A disorder in the function of GATs may contribute to AD pathology depending on increased or decreased receptor function. Finally glia cells especially astrocytes are of vital importance to proper functioning of the GABAergic system. Astrocytes are also able to express both GABA receptors and GABA transporters, and are able to synthesize GABA itself (Buğra EK18). Armed with all the necessary GABA elements, astrocytes are able to heavily regulate GABAergic transmission through various mechanisms [77]. With its crucial role in regulating neurotransmission, astrocytic dysfunction is heavily implicated in AD pathology [78].

First, nearly all elements of GABAergic transmission are affected structurally, especially GABA receptors. For example, the concentration of neurotransmitter GABA itself is aberrant in AD temporal complex [79]. For the receptors, animal model study associated with AD examined the structural changes of GABA receptors. Examining the reduction of post and presynaptic GABA_B_ receptors on the neuronal surface of hippocampal CA1 pyramidal cells in a mouse model detected a severe reduction in surface GABA_B_ receptors [80]. Further investigating the nano-sized changes in GABA_B_ receptors and G protein-coupled inwardly rectifying potassium channels (GIRK) in the hippocampus region of APP/PS1 mice showed that there was the spatial interaction between the two-dimensional distribution of GIRK channels and GABA_B_ receptors. Immunogold SDS-FRL technique study reported that GIRK1 and GIRK2 channels were significantly reduced on the neuronal surface and axon terminals of CA1 pyramidal cells, and the co-clustering of GABA_B_ receptors and GIRK channels decreased and dissipated [81] (Fig. 1B). On the other hand, GABA_A_ receptor subunit expression in the hippocampus, subiculum, entorhinal cortex, and superior temporal gyrus regions revealed that the expressions of GABA_A_ receptor subunits had region- and layer-specific effects from [82] AD [83]. Similarly, in another experiment on loss of functional GABA_A_ receptors in AD, mRNA levels of α1, α2, β2, β3, α5, γ2, and δ subfamilies were significantly decreased compared to the control group [84]. Another study investigated the damaged expression of GABA signaling components showed that individuals with AD were found to have a significant decrease in the expression of various GABA subunits in the middle temporal gyrus (MTG) region [85]. AD-related structural damage is not limited to GABA receptors even whole morphology of the GABAergic interneurons, which are responsible for the bulk of the GABA supply through the brain, are affected [86]. On the Knock-in AD model again, effects of Aβ on the hippocampal interneurons was examined. Significantly abnormal dystrophic axon terminals were observed in PV + interneurons [87]. Although PV neurons are relatively morphologically resistant to AD-related changes, another study reported that PV + IN projections are significantly increased in the CA1 and CA3 region of the APP/PS1 mice [88]. Structural abnormalities observed in AD are not without functional consequences which are heavily investigated for the intention to develop an effective and safe treatment system for AD. For example, in the study just mentioned, a respective desynchronization of consummatory behavior associated with SPW-R and high functional behaviour associated with y-osciliations between CA3 and CA1 regions was observed in APP/PSS1 mouse [88]. Additionally, another study conducted on APPNLF mice reported decreased fast-large sIPSCs in the temporal region [89]. The reported depression of fast-large sIPSC could be a result of a dysfunction in PV + IN GABAaR [89] which generates a similar type of conductance. In support with this assumption relating to involvement of GABAaR, another study reported a decreased expression of GABAAr-mediated tonic conductance in AB42 injected C57BL/6 animal model [90]. An answer for the underlying cause of disruptions in GABA-related conductance is complex and multifactorial however glial neuroinflammation is a strong trigger. In support with this argument, another study investigated the relationship between glial activation and KCC2 which is a K + Cl– co-transporter crucial for inhibitory function of GABA A receptors in hippocampi APP/PS1 mice. In the study, glial activation by Aβ and subsequent inflammatory response significantly decreased expression of in GABA AR receptors and KCC transporters [91]. The observed downregulative effect of inflammation was apparently mediated by BDNF. Considering the fact BDNF is a vital factor for proper functioning of GABAa-related anion transporters, the reported findings may be pointing to another potential mechanism for disruption in neural transmission [91]. Another study observing the CA1 region of the APP/PS1 mice confirmed the importance of BDNF in AD pathology. The AD mice showed a drastic impairment in the ability to cleave proBDNF to BDNF [92]. Additionally, external BDNF injection with proBDNF inhibitor attenuated the reduced expression of KCC2 and Cl conductance [92]. Collective evidence suggests that for AD pathology at the least, GABA A-related mechanisms are much more dominant than GABAb receptors. However several studies revealed a critical function for GABAb receptors that may be important for AD pathology. A study reported that APP, the precursor protein for Aβ, is able to complex with Sd1 domain of GABAB receptors and by complexing with GABAb receptors, APP is able regulate the axonal traffic of the GABAB receptor [93]. As for the consequences of APP/GABAb relating to receptor function itself, literature presents a contradiction. While an earlier study reported that APP takes part in GABAergic synaptic transmission [94], a novel study found no significant connection [95]. However, it is at the very least possible to suggest that forming of APP/GABAb complexes prevents APP from cleaving into pathologic Aβ formations [93]. How APP/GABAb interactions affect and in turn get affected by AD pathology is yet to be illuminated. Astrocytic GABAergic system is also profoundly altered in AD pathology and is being heavily investigated as a source of alternative treatment. Recent studies have reported that there is a significant disruption in the astrocytes ability to metabolizing or transferring glutamine to neurons even in the very early stages of the AD [96, 97]. Considering the fact that neurons depend heavily on the astrocytic glutamine, the reduction in the astrocytic glutamine metabolism has at least several consequences [98]. Firstly, as GABA is synthesized from glutamine, initially GABA synthesis is reduced in neurons resulting in a significant increase of spontaneous excitatory post synaptic potential sEPSC [97]. Secondly some potential compensatory mechanisms take place. The nature of these compensatory mechanisms was observed in 5xFAD mice; neural glutamine metabolism is increased probably to make up for the deficient glutamine supply from astrocytes [96]. Another important observation reported in a study investigating astrocyte GABAergic system effects of astrocytic GABA transporter 3/4 was investigated in the AD Knock-in model. Elevated levels of the astrocyte-specific GAT 3/4 was observed in both CA1 and DG regions in mouse model AD. Elevated expression of GAT 3/4 was correlated with enhanced tonic inhibition and, in parallel with other observations, elevated baseline spontaneous synaptic excitation was observed [99]. It is important to note however the results from the knock-in model is not in line with an earlier post-mortem human ad brain study in which decreased expression of GAT transporters was reported [100], although GAT investigated in human study was not astrocytic specific [100]. The causal relationship between GAT transporters and impaired astrocytic metabolism is not fully clear. Interestingly, in a study on APP/PS1 mice, it was observed that astrocytes closer to amyloid plaques are more reactive and more GABA-dense [101]. However, at the end-stage phase of the disease, GABA levels were similar to the pre-plaque stage and astrocytic soma size was significantly larger. It is possible that as the soma size increases, GABA is released aggressively from the astrocytes which would cause drop in astrocytic GABA levels as another study reported a decrease in memory function of mice was due to excessive GABA production by astrocytes around amyloid plaques [102]

Thanks to the studies aiming to illuminate the pathologic mechanism several drugs that are targeting the various GABAergic mechanisms. For example, studies have shown the beneficial effects of drugs targeting the GABAergic system. In one study involving 5xFAD AD mice, a greatly correctable decreased inhibitory synaptic transmission was improved in the early period with the GABAaR agonist, gaboxadol (GBX) [103]. In another pharmacological approach, the APP-PS1 mouse model was pharmacologically treated with Artemisinin at the pre-plaque time, resulting in an increase expression and phosphorylation of coat protein Gefrin on inhibitory synapses and an increase in the expression of GABAaR γ2 receptors [104]. APP/PS1 animal model exposure to gamma waves showed that amyloidogenic processing of gamma irradiation was reduced by fixing amyloid precursor protein (APP) to the cell membrane. In addition, with the interaction of APP located on the cell membrane with K + /Cl– cotransporter (KCC2), the levels of AD-bound GABAaR a1 approached the physiological value [105]. Pharmacological targeting of astrocytes is also a possible treatment approach. KD-S2010 is a novel reactive astrocyte GABA synthesis inhibiting MAO-B inhibitor. In the animal study for KD-S2010, it was reported that the compound is able restore spatial learning and memory both in short-term and in long-term treatment which is a significant improvement over other MAO-B inhibitors [106]. To summarize AD pathology, especially amiloid load is associated with structural and functional abnormalities which could range from cellular morphological alterations to severe electoral desynchronizations and disruption ultimately resulting in cognitive deficits (Table 2).

**Table 2:** The role of GABA in AD based on previous literature

Study type	AD model	Drug and dose	Observation	Remarks	References
In vivo	APP/PS1	–	Drastic reduction in post and presynaptic GABAβ receptors on the neuronal surface of hippocampal CA1 pyramidal cells was detected	Interventions to achieve membrane stabilization of GABAβ receptors could be a potential way treatment for AD	[81]
In vivo	APP/PS1	–	GIRK1 and GIRK2 channels were significantly reduced in the neuronal face and axon terminals of CA1 pyramidal cells. Disruptions in the co-aggregation of GABAβ receptors and GIRK channels have been reported	It may be possible to say that the GABAergic system is affected by AD pathology, not only structurally but also functionally	[80]
Human study	Post-mortem human brain tissue	–	It has been reported that the expression of subunits of GABAa receptors in the hippocampus, subiculum, entorhinal cortex, and superior temporal gyrus regions are region and layer specifically affected by AD	Observed results may be a natural compensatory response rather than a direct pathological effect of AD	[84]
Human study	Post-mortem human brain tissue	–	It has been found that individuals with AD have a significant decrease in the expression of various GABAα subunits in the MTG region	GABAa subunits may be altered in AD pathology alternatively there may have been a massive loss of GABAergic neurons	[85]
In vivo	APP/PS1	–	A significant reduction in memory function of mice has been reported as a result of excessive GABA production by astrocytes around amyloid plaques	A good understanding of the relationship between astrocytes and the GABAergic system in AD pathology may be a source of alternative treatment	[102]
In vivo	5XFAD	GBX (0.25 μL/h for 28 days)	Decreased inhibitory synaptic transmission was detected after the treatment with GABAaR agonist GBX	GABAergic system enhancement may be an effective therapeutic approach to AD	[103]
In vivo	APP/PS1	Artemisin (10–100 mg/kg)	Increased expression of Gefrin and GABAaR y2 and increased gefrin phosphorylation have been reported	Changes in early AD may be compensatory in nature	[81]
In vivo	APP/PS1	Gama-ray oscillation (40 Hz)	After light therapy, GABAaR a1 levels approached the physiological value	Gamma radiation may be a drug-free, non-invasive treatment for AD	[105]
In vivo	APP^NL−F/NL−F^	–	Decreased GABAergic function has been reported as a result of positive allosteric modulation of GABAaRs	Positive GABAaR modulators may play an important role in the early treatment of AD	[83]
In vivo	–	–	–	–	[88]
In vivo	APP^NL−F/NL−F^	–	Significant GABAergic dystrophic axon terminals in PV + IN was observed	Better understanding of how Aβ accumulation leads to hallmarks of AD pathology may be crucial for an effective treatment	[87]
In vivo	APP/PSS1	–	The APP/PS1 animals exhibit a large increase in PV + IN projections in the CA1 and CA3 areas. Additionally, there was evidence of a desynchronization of consummatory behavior related with SPW-R and high functional behavior linked to y-oscillation between the CA3 and CA1 regions	The results highlight the critical significance of structural modifications of PV + interneurons throughout the early state of Aβ overproduction in AD, preserving initially minor abnormalities at the network level, and promote a compensatory upregulation of hippocampus inhibitory GABAergic terminals	–
In vivo	APP^NL−F/NL−F^	–	A decrease in the fast-large sIPSCs in temporal region of AD mice was reported	The drop in the overall average amplitude of the sIPSCs in AppNL-F mice was driven by a decrease in the average amplitude of the big sIPSCs but not the small ones,	[89]
In vivo	APP^NL−F/NL−F^	–	Elevated levels of the astrocyte-specific GAT 3/4 was observed in both CA1 and DG regions in mouse model AD. Enhanced tonic inhibition and elevated baseline spontaneous synaptic excitation was measured	A good understanding of the relationship between the astrocyte GABAergic system in AD pathology may be a source of alternative treatment	[58]
In vivo	C57BL/6	–	A1-42 hippocampal injection is sufficient to cause an increase in inhibitory GABAergic tonic conductance in the CA1 area of the mouse hippocampus, which is mediated by extrasynaptic type A GABA receptors	GABAARs may have a substantial influence on hippocampus shape and function, disrupting excitatory and inhibitory balance and perhaps leading to cognitive impairments in AD	[90]
In vivo	APP/PS1	–	GABAaR receptor and KCC transporter expression was dramatically reduced after glial activation by Aβ and subsequent inflammatory response	Soluble Aβ may affect GABA inhibition via regulating KCC2 levels especially in earlier stages of AD. Additionally KCC2 might be used as a biomarker for Alzheimer’s disease	[91]
In vivo	APP/PS1	–	The capacity of the AD mice to cleave proBDNF to BDNF was severely impaired. Furthermore, exogenous BDNF injection with a proBDNF inhibitor inhibited the expression of KCC2 and Cl conductance	Hippocampal proBDNF/BDNF signaling is capable of regulating GABAergic transmission and, as a result, plays an important role in the development of cognitive impairment in AD animal models	[92]
In vivo	*GB1a*^*−/−*^*, GB1b*^*−/−*^*,* and *GB2*^*−/−*^ mice with BALB/c background, *APP*^*−/−*^ mice with C57BL/6 background	–	By complexing with GABA receptors, APP can interact with the Sd1 domain of GABAB receptors. APP may control the receptor’s axonal flow	The formation of APP/GABAb complexes preventing APP from cleaving into pathogenic Aβ forms	[93]
In vivo	5xFAD	–	Data show that impaired astrocyte metabolism reduces glutamine synthesis, which directly impairs neuronal GABA production in the 5xFAD brain	The abnormalities of synaptic excitation and inhibition in the AD brain may be caused by astrocyte metabolic collapse	[96]
In vivo	5xFAD	–	Reduced neuronal GABA production lead to a significantly increased spontaneous excitatory post synaptic potential		[97]
Human study	Post-mortem AD brain tissue	–	Layer and region specific dysregulations of BGT-1, GAT1, and GAT3 was reported	These findings reveal that GAT expression varies by brain area and layer in Alzheimer’s disease	[100]
In vitro	APP/PS1	–	Astrocytes around amyloid plaques were shown to be more reactive and GABAdense, and as the illness progressed, GABA levels was comparable to pre-plaque values	It is possible that when the soma size rises, GABA is aggressively released from the astrocytes, resulting in a considerable drop in GABA concentration	[101]
In vitro	APP/PS1	KD-S2010 10 mg/kg/day	It has been shown that the KD-S2010 may restore spatial learning and memory in both short-term and long-term therapy, which is a substantial improvement over other MAO-B inhibitors	According to the findings, MAO-B is an important upstream molecular actor in astrogliosis. Furthermore, these findings show that KDS2010 might be a viable pharmaceutical approach for preventing astrogliosis and, as a consequence, alleviating cognitive symptoms seen in Alzheimer’s disease	[106]

*AD* Alzheimer’s disease, *GABAaR* GABAa receptor, *MTG* middle temporal gyrus

## Monoamine-based Neurotransmitters

### Dopamine

Dopamine (DA) is a monoamine neurotransmitter that has a role in higher function performance of the brain. Basically, it has a neural effect on hormonal disorders associated with AD [107]. The cognitive ability to control behaviors proportional to goals is managed by DA residing in the prefrontal cortex (PFC). The known mechanisms of action of DA in the PFC are controlling emotional input, maintaining and manipulating the working memory mechanism, as well as transmitting motor commands [108]. Although DA mediates reward and motivation, it has been observed that DA systems in the brain also have a regulatory effect on the chronic pain center [109]. Dopamine 1 receptor (D1R) and dopamine 2 receptor (D2R) are both involved in cognitive function [108]. D1R is located in the CNS and has a peripheral mechanism of action on motivation, cognitive function, and blood pressure. D1R also has effects on the development of human neoplasms, neoplastic cell proliferation, autophagy, apoptosis, and enrichment of the cancer stem cell population by regulating signaling pathways of DA [110]. Additionally, a link was also observed between the changes recorded in cognitive performances and the D1R, where D1R has been found to have a regulatory effect on transcriptional activity [111]. The effect of D2R in various cell types at the developmental period varies. Accordingly, over-stimulation or over-expression of D2R is one of the determining factors in triggering neurophysiological diseases [112]. In patients with AD, behavioral and psychological manifestations of dementia may be caused by dysfunction in the D2R metabolism in the striatal basal ganglia nucleus 62 [113]. Testing striatal D2 receptor density in AD-related disorders may indeed provide a facilitating approach for future studies (Table 3).

**Table 3:** The role of dopamine in AD based on previous literature

Study type	AD model	Drug and dose	Observation	Remark	References
In vitro In vivo	APP/PS1 mice, NMDA excitotoxic cell	PEI (5 ml/mg cDNA of 10 mM PEI)	The heteroreceptor has been found to reduce NMDA-mediated excitotoxic cell death	D1DR-containing heteroreceptors can reduce cell death in AD-related patients	[76]
	Twelve-month-old female 3xTg-AD and WT mice (*n* = 4/genotype)	–	Lack of tau accumulation and low Aβ storage in the hippocampus was due to DAergic dysfunction	Detection of DAergic dysfunction may shed light on future therapeutic approaches for AD	[114]
In vivo	Heterozygous Tg2576 mice	DOPA (10 mg/kg), benserazide (12 mg/kg)	VTA DAergic neuron degeneration caused loss of function in CA1 synaptic plasticity	DA has an effect on the loss of consciousness in AD	[115]
	Male APP/PS1 mice	DMSO (3–10 mg/kg, i.p.), L-SPD (10 mg/kg, i.p.)	Prevention of synaptic loss by activating the L-SPD, DRD1/PKA signaling pathway was mediated by PKA	It may be investigated as a therapeutic drug to prevent L-SPD AMPA receptor trafficking	[116]
In vivo	FAB mice model (*n* = 12)	MK-801 (0.75 µg/kg, ip)	It has been determined that the main reason for the treatment of comorbid psychosis in AD is the disorder in the dopamine system	Abnormal regulation of the dopamine system may cause comorbid psychosis in AD	[117]
In vivo	C57/BL6 male mice (n = 30)	LSD (5–160 µg/kg, intraperitoneal)	LSD has a role in controlling the activity in the CSTC circuit	Haloperidol induces neuronal activity by increasing burst-fire activity in reticular thalamus. In conclusion, LSD has an effect on consciousness activities in humans	[118]
Human study	AD model of human (*n* = 30)	RTG (4 mg per week), RVT (4.6 mg) PLC (transdermal patch)	Restoring the altered mechanisms of LTP was accomplished by DA agonists	DA may be used in new treatments based on DAergic stimulation	[119]
	Human with AD (*n* = 10)	–	D2DR disorder in the striatal caused behavioral abnormalities	Triatal D2 receptor may be used in AD-related disorders	[120]
In vitro, human study	Brain tissue from the superior temporal gyrus, Caucasian cases (*n* = 727)	–	DRD1 has been found to have a regulatory effect on transcriptional activity	A connection may be made between DRD1 and conscious performances	[121]

*DRD1* dopamine 1 receptor; *WT* wild type; *i.p* intraperitoneally; *AD* Alzheimer’s disease; *RTG* dopamine agonist rotigotine; *VTA* ventral tegmental area; *DA* dopamine; *L-SPD-AMPA* l-stepholidine α-amino-3-hydroxy-5-methylisoxazole-4-propionic acid; *LTP* long-term potentiation; *D2DR* dopamine 2 receptor, APP; *PEI* polyethylenimine; *DMSO* dimethyl sulfoxide; *PKA* protein kinase A; *Aβ* amyloid beta; *MCI* mild cognitive impairment; *NMDA* N-methyl-d-aspartic acid; *RVT* rivastigmine; *PLC* placebo; *LSD* psychedelic lysergic acid diethylamide; *CSTC* cortico-striato-thalamo-cortical

DA are flavoenzymes catalyzed by the oxidative deamination of various neurotransmitters, including other amines such as norepinephrine, tyramine, and serotonin, where the resulting deamination causes the release of harmful by-products such as ammonia, peroxides, and aldehydes. The changes in concentration of biochemical neurotransmitters in the brain triggered by monoamine oxidase (MAO) may be directly related to various neurological disorders such as AD and PD. MAO inhibition has a general anti-Alzheimer’s effect as a result of the reduction of oxidative stress activated by MAO enzymes [122]. Patients with Alzheimer’s disease had significantly lower dopamine levels compared to controls (SMD =  − 1.56, 95% CI − 2.64 to − 0.49). Additionally, dopamine 1 receptor (SMD =  − 5.05, 95% CI − 6.14 to − 3.97) and dopamine 2 receptor (SMD =  − 1.13, 95% CI − 1), levels were found to be lower in patients with AD, when compared to normal individuals are indicated. As a result, the dopaminergic system has been found to be associated with the progression of AD [123]. According to a study conducted on DA neurons in 2018, DA subpopulations were found to regulate motivational behaviors. Controlled by afferent inputs, mesolimbic dopamine (DA) neurons play a central role in reward processing. Neurons in the lower part of the medial shell of the nucleus accumbens (NAc) have an inhibitory role on two distinct populations of mesolimbic DA neurons. Accordingly, NAc lateral shell neurons primarily synapse on the ventral tegmental area (VTA) GABA neurons, causing disinhibition of DA neurons that reflect back to the NAc lateral shell [124]. Consequently, there may be an inhibitory distinction between subtypes of mesolimbic DA neurons. The cause of DAergic system failure in AD is not fully explained. An age-related loss of DAergic deficits, but a significant loss of DAergic formations in the nigra pars compacta (SNpc) and VTA, has been observed. Particularly in the Tg2576 toll model, studies occurring in the midbrain DAergic region indicate that degeneration of the VTA system causes lower DA output in the hippocampus and nucleus accumbens (NAc) shell. The resulting progression of DAergic cells consists of impairments in CA1 synaptic plasticity, and memory performance.

Degeneration of dopaminergic neurons in the VTA causes memory deficits and loss of consciousness in AD [115]. Tau NFT accumulation and low Aβ storage deficiency in the hippocampus have been observed at an early age in 3xTg-AD mouse model, resulting in dopaminergic dysfunction [114]; the main reason for the treatment of comorbid psychosis in AD is the disorder in the dopamine system. Accordingly, considering that it is a key pathology area in AD, abnormal regulation of the dopamine system causes comorbid psychosis in AD. Abnormal hippocampal activity was detected in a mouse-to-mouse study containing ferrous amyloid butionine (FAB) in 2023 [117]. In conclusion, abnormal regulation of the dopamine system may cause comorbid psychosis in AD. Based on the findings of imaging studies, it has been determined that psychedelic lysergic acid diethylamide (LSD) has a role in controlling the activity in the cortico-striato-thalamo-cortical (CSTC) circuit. Accordingly, the dopamine D2 receptor (D2) antagonist haloperidol administered after LSD induces neuronal activity by increasing burst-fire activity in reticular thalamic neurons inhibited by LSD [118]. In conclusion, it can be said that LSD has an effect on consciousness activities in humans.

**Fig. 2:**
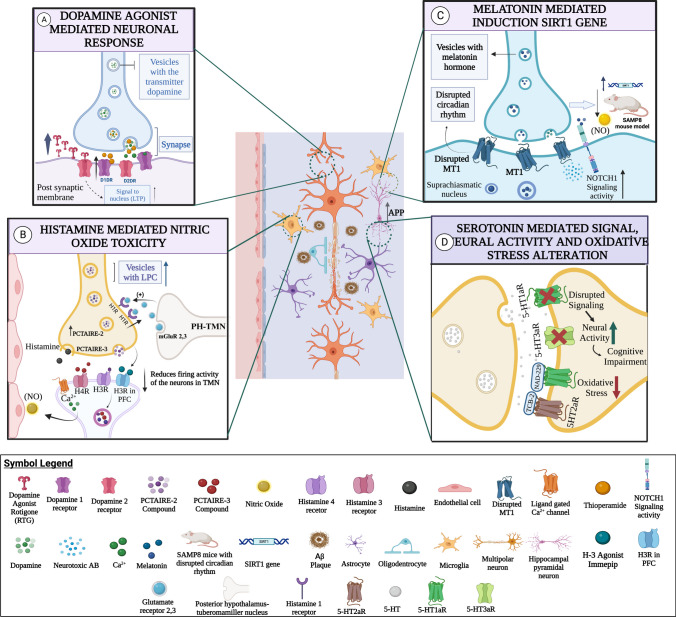
**A** Dopamine agonist mediated neuronal response. High concentration of dopamine agonist rotigotine (RTG) inhibits local damage in plasticity. RTG restores altered mechanisms of LTP-like cortical plasticity. Thioperamide, an antagonist of histamine-3 receptors, has been observed to increase the firing activity of dopamine neurons in the ventral segmental area. Accordingly, it can be said that histamine antagonist, one of the brain monoamines, plays a role in the treatment of cognition. **B** Histamine mediated nitric oxide toxicity. Increased intracellular concentration of histamine stimulates the production of nitric oxide (NO) from endothelial brain cells by increasing Ca2+ release. When H3Rs expressed in the tuberomammillary nucleus (TMN) and PFC of the hypothalamus were examined, it was determined that the H(3)/H(4)-agonist, thioperamide decreased the firing activity of neurons. It can be concluded that H3Rs, and especially those expressed in the PFC, play an important role in the autoregulation of histamine neurotransmission. Histaminergic activity is regulated by group II metabotropic glutamate receptors (mGluR 2 and 3) using systemic dosing with mGluR 2/3 agonists and antagonists and an mGluR 2 positive allosteric modulator. In addition, it increased the positive cleavage and release of glutamate neurons of the histaminergic system in the histaminergic cell bodies in PH-TMN and in the neurons projecting to PH-TMN. **C** Melatonin mediated induction SRT1 gene. Degeneration occurring in MT1 receptors causes circadian rhythm disorder due to melatonin hormone. SIRT1 gene expression induced by melatonin reduced neurotoxic Aβ accumulation and oxidative stress. In a study on the cerebral cortex, it can be said that melatonin application has a healing effect on the Notch1 signaling pathway. **D** The role of serotonin in AD. Upregulation of the 5-HT4 receptor in the early phase of AD reduces Aβ accumulation. At the same time, 5-HT7 receptor activation reduces apoptosis in the hippocampus. Impaired 5-HT1aR and 5-HT3aR signals can lead to cognitive disorders. In addition, the low functioning of SERT carriers can cause cognitive disorders

Possible treatments for AD through the dopaminergic system have been suggested. For example, prevention of loss of synaptic plasticity and memory in AD models by l-stepholidine (L-SPD) have been investigated where L-SPD activates the D1R/PKA signaling pathway through protein kinase A (PKA) [116]. Thus, L-SPD may be investigated as a potential therapeutic agent for AD, as it may prevent α-amino-3-hydroxy-5-methylisoxazol-4-propionic acid (AMPA) receptor trafficking by activating the D1/PKA signaling pathway. Besides that, DA agonists were able to restore altered mechanisms of long-term potentiation (LTP)-like cortical plasticity and inhibit damage caused by plasticity by preventing regional loss [119] (Fig. 2A). One study showed that D1R-containing heteroreceptors have a promising therapeutic effect in preventing cell death in AD-related patients, through mediation of NMDA excitotoxicity. By bioluminescence resonance energy transfer (BRET), D1 or H3 receptors have been observed to form heteromers with NR1A/NR2B NMDA receptor subunits. Consistent with allosteric receptor-receptor interactions, H3 receptor antagonists reduced NMDA- or D1 receptor-mediated excitotoxic cell death in cortical organotypic cultures. However, H3 receptor antagonists reversed the toxicity caused by ß1-42-amyloid peptide. Based on the study’s conclusion, histamine H3 receptors in D1-H3-NMDA heteroreceptor complexes emerge as promising targets to prevent neurodegeneration [120]. Likewise, cerebral dopamine neurotrophic factor (CDNF), one of the proteins that regulate neuronal plasticity, may also be used as a therapeutic agent, especially in blocking dopaminergic decline or restoring the function of damaged neurons. This agent acts mainly through the transmission of CDNF to the brain parenchyma, protecting DA neurons and inhibiting its numerical decline [121]. Therefore, these evidences suggest the potential of the dopaminergic system as a therapeutic target for AD.

### Norepinephrine

Norepinephrine (NE), or noradrenaline, is a neurotransmitter that belongs to the catecholamine family. NE is predominantly synthesized by noradrenergic neurons in the locus coeruleus (LC) region of the brain stem [125]. The effect of loss of LC neurons in the early stage of AD have been reported by transgenic AD mouse model, in which LC destruction in the pre-plaque stage caused aggravation of cognitive damage [126]. NE itself is involved in cognitive processes including cognitive flexibility and active memory [125, 127].

Norepinephrine is synthesized in neurons via the amino acid tyrosine. Tyrosine is first converted to DOPA and then to dopamine. Subsequently, dopamine is converted to NE by the enzyme b-hydroxylase [128]. NE, which is synthesized and released into the synaptic space, exerts its effects on post- and presynaptic adrenergic receptors (AR). ARs are G protein-associated receptors and nine ARs have since been identified. These nine AR receptors were A1 (A1a, A1b, A1d), A2 (A2a, A2b, A2c), and B (B1, B2, B3) [129]. A1 ARs are believed to generally mediate an excitatory response [129]. Pharmacological and genetic dissection of A1 ARs have demonstrated that the A1 family has a major impact on memory functions and synaptic plasticity [130–132]. Post- and pre-synaptic A2 ARs, on the other hand, are involved in the neurotransmitter regulation of the CNS [133, 134]. A2 AR antagonism may negatively affect neuronal excitability and learning function in a dose-dependent manner [135, 136]. A2 ARs have been discovered to be involved in the SorLA-dependent (a receptor that hinders the transformation of amyloid precursor protein (APP) into soluble APP and amyloid-beta peptide in cultured neurons) endocytic regulation of APP. A2 ARs reduced SorLA-dependent regulation of mature APP sorting by interfering with the co-localization and contact between SorLA and mature APP with G protein-associated signaling [137]. The results may indicate that A2 ARs, a previously under-appreciated therapeutic target, are of particular importance for the treatment of AD. On par, in another study, CD1 mice A2 receptors are antagonized by 2-pentadecyl-2-oxazoline (PEA-OXA), a natural compound. Repeated PEA-OXA treatments improved social behavior and cognitive function in sA-injected mice and partially reversed the sAB-induced LTP deficit in hippocampal DG [138]. On the basis of these findings, PEA-OXA may be regarded as a unique substance for upcoming ground-breaking treatments for dementia or AD, for which the current medications are ineffective. Although β-adrenergic receptors (B ARs) are mainly expressed in organs outside the nervous system, B ARs have also been detected in the brain [139] and may have a role in memory function [140, 141]. Indeed, upon activation of ADRB2 receptors on an AD animal model, ameliorated mitochondrial dysfunction induced by Aβ and attenuated mitophagy deficits via the ADRB2/Akt/PINK1 pathway was reported [142]; findings indicate that ADRB2s may be a promising therapeutic target for AD. Considering the effect of B ARs on memory, there seems to be a possible relationship between AD characterized by severe memory impairment. The increase in AR function may have significant effects on cognitive performance in people with early-stage AD as well (Table 4). Thus, due to the wide range of cognitive effects of ARs, the NE concentration in the synaptic vesicle should be tightly regulated for healthy neuronal activity and may have a strong implication in AD development and progression. An answer to why cognitive damage aggravates could be found in the electrophysiology of LC as investigated in a study done on mice and human tissue. In the said study, it was observed that accumulation Aβ oligomers inhibits the GABAa3 receptors which results in a hyperexcitable LC [126]. The evidence points to a strong relationship between A-O and 3-GABAARs in the LC of Alzheimer’s patients and suggests that these receptors may be able to dysregulate LC activity. Dysregulated noradrenergic electrophysiology is also observed outside of LC. A study investigated cerebellar activity on TgCRND8 AD mice. Gathered data indicated that the cerebellum of 2-month-old Tg mice shows a severe impairment of the noradrenergic regulation of the Parallel Fiber-Purkinje Cell synapse [143]. This work reveals that one of the initial neuroanatomical impacts of APP overexpression alongside LC is cerebellar circuit disruption. In line with these results, another study investigated the noradrenergic system on human-tau expressing mouse shortening the gap between human and animal studies. The findings imply that tau buildup in the LC and the modifications in noradrenergic firing patterns that ensue may also play a role in depressed behaviors in human tau animals. Additionally, anxiety-like behaviors were seen at the 6-month mark, which might be the result of LC neurons losing function or experiencing neuronal death [144]. To establish a causal link between LC disease and AD symptoms, more research in AD mice models is required.

**Table 4:** The role of Norephinephrine in AD based on previous literature

Study type	AD model	Drug and dose	Observation	Remarks	References
Human study, in vivo	Post-mortem human brain tissue/B6C3-Tg animal model	–	Neuronal hyperexcitability and altered brain NA levels were detected in the LC in the presence of Aβ oligomer	Clinical screening of LC may become a common practice in the early diagnosis of AD	[145]
Human study	Retrospective study	–	A significant and positive correlation was found between NE level and CSF AB_1-42_ level	Plasma NE deregulation and excessive LC activation may be involved in the diagnosis and follow-up of AD	[146]
Human study	Clinical Trial	Atomoxetine (weekly increments after 10 mg/kg on first day)	Atomoxetine has been associated with significantly reduced CSF tau levels compared to placebo	Atomoxetine may play a role in clinical practice in improving LC function	[135]
In vivo, human study	APP-PSEN1, post-mortem human brain tissue	–	Accumulated Aβ oligomers inhibited the GABAa3 receptors which resulted in a hyperexcitable LC	The evidence points to a strong relationship between A-O and 3-GABAARs in the LC of Alzheimer’s patients and suggests that these receptors may be able to dysregulate LC activity	[126]
In vivo	TgCRND8	–	Cerebellum of 2-month-old Tg mice showed a severe impairment of the noradrenergic regulation of the parallel fiber-Purkinje cell synapse	One of the initial neuroanatomical impacts of APP overexpression alongside LC is cerebellar circuit disruption	[143]
In vivo	C57BL/6 mice, htau ± mice	–	tau buildup in the LC and the modifications in noradrenergic firing patterns that ensue play a role in depressed behaviors in human tau animals	To establish a causal link between LC disease and AD symptoms, more research in AD mice models is required	[144]
In vivo	C56BL/6 J mice	Clen (2 mg mg/kg/day)	Activation of ADRB2 ameliorated mitochondrial dysfunction induced by Aβ and attenuated mitophagy deficits via the ADRB2/Akt/PINK1 pathway	ADRB2s may be a promising therapeutic target for AD	[142]
In vivo	CD1 Mice	2-Pentadecyl-2-oxazoline (PEA-OXA) (10 mg/kg)	Repeated PEA-OXA treatments improved social behavior and cognitive function in sA-injected mice and partially reversed the sAB-induced LTP deficit in hippocampal DG	PEA-OXA may be regarded as a unique substance for upcoming ground-breaking treatments for dementia or AD, for which the current medications are ineffective	[138]
In vivo	APP23	Prazosin (Daily 1 mg/kg for 2 weeks)	Long-term use of prazosin slowed the course of AD by increasing astrocytic proliferation, release of anti-inflammatory cytokines, and production of APOE	AD’in erken dönemindeki değişiklikler kompansatif değişiklikler olabilir	[147]
In vivo	FADX5	Avenanthramide-C (6 mg/kg for 2 weeks)	Avn-C, a natural compound, has been reported to have anti-AD properties	The fact that Avn-C does not cause side effects and is a natural compound found in oats may make it a frequently preferred compound in the treatment of AD	[148]
In vivo	APP/PS1	–	SorLA-dependent regulation of mature APP sorting was reduced by A2′ARs interfering with co-localization and contact between SorLA and mature APP by G protein-associated signaling	Contrary to popular belief, A2-ARs may show a high therapeutic potential	[137]
In vivo	APPV717I-APP/PS1	–	The study found that chronic NE depletion in the mice led to increased neuroinflammation in areas innervated by the locus ceruleus (LC), resulting in impaired microglial phagocytosis and recruitment to Aβ plaques. NE was shown to positively regulate microglial Aβ clearance and suppress neuroinflammation. Additionally, NE depletion was associated with reduced Aβ inclusion within microglia	The findings suggest that NE plays a crucial role in regulating microglial functions and Aβ clearance in AD. Restoration of brain NE levels, potentially through the use of NE precursors, may support therapeutic strategies targeting microglial function in AD. The study also highlights the potential of NE-targeted therapies, such as vaccination against Aβ, in the context of NE depletion	[149]
In vivo	APP/PS1	–	APP/PS1 mice’s cortex, hippocampus, and spinal cord showed higher levels of microglial activation and inflammatory cytokine production as compared to healthy mice. AD animals showed a greater degree of LC-NE neuron and fiber loss as well as decreased norepinephrine transporter (NET) expression	The findings imply that substantial LC-NE system degradation is correlated with increased neuroinflammation and microglial activation in the brain and spinal cord of APP/PS1 animals	[150]
In vivo	C57BL/6 J with different mutations	Antagonist (Varying doses): Metoprolol, CGP-20712A, Atenolol Agonists (Varying Doses): Xametorol, Mabuterol	Beta blockers both potentiated LPS induced systemic infection and APP induced CNS inflammation	The data that was gathered points to beta-blockers’ proinflammatory functions in the peripheral during systemic inflammation and in the central nervous system during pre-existing neuroinflammation	[151]

*AD* Alzheimer’s disease, *AB* amyloid beta, *BOS* cerebrospinal fluid, *NE* norephinephrine, *LC* locus courelus, *bAR* beta adrenergic receptor, *A1-AR* alpha 1 adrenergic receptor, *A2-AR* alpha 2 adrenergic receptor, *AvnC* aventhramide C, *CNS* central nervous system, *NET* norepinephrine, *SorLA* sorting-related receptor with a repeat, DG dentate gyrus, *LTP* long-term potentiation

The reuptake of NE is regulated by the norepinephrine transporter (NET). Dysfunction of the NETs has been closely associated with psychiatric disorders including attention-deficit hyperactivity disorder (ADHD) and major depressive disorder [152, 153], and therefore may also play a role in AD. In one study, a significant positive relationship was found between plasma NE levels and cerebrospinal fluid (CSF) Aβ _1–42_ levels [145]. Plasma NE de-regulation and excessive LC activation may be involved in the diagnosis and follow-up of AD.

**Fig. 3:**
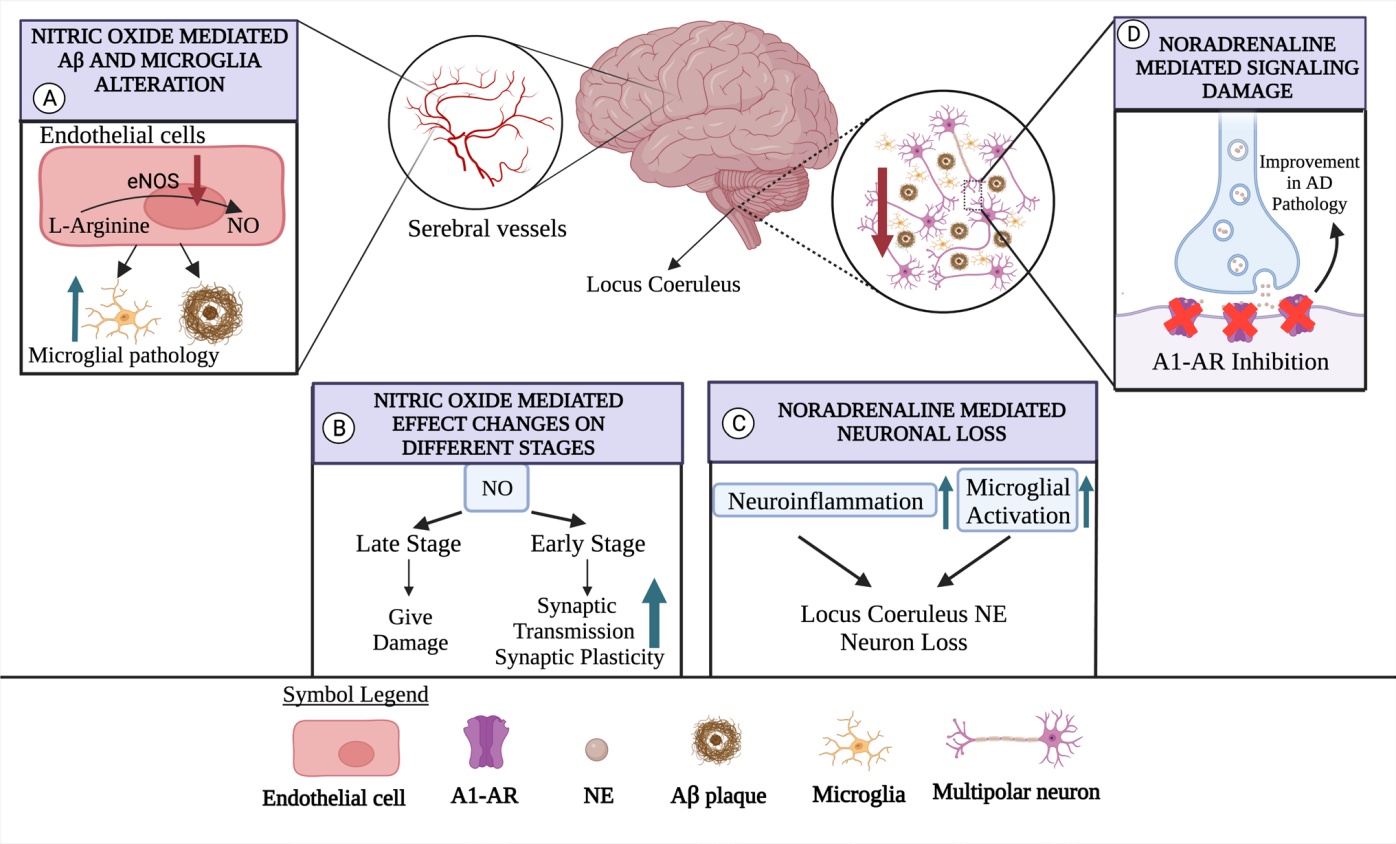
**A** The role of nitric oxide in AD. As a result of eNOS deficiency in endothelial cells in the walls of brain blood vessels, NO production is low. This increases Aβ brain accumulation and microglial pathology. **B** The role of nitric oxide in AD. NO increases synaptic transmission and plasticity in the early stages of AD. However, as the disease progresses, NO can cause major damage to brain cells. **C** The role of noradrenaline in AD. Elevated neuroinflammation and microglial activation in the brain is accompanied by the loss of LC-NE neuron. **D** The role of noradrenaline in AD. In AD, neurons in the first locus coeruleus are lost. When the A1-AR signals of neurons in the locus coeruleus and CNS are inhibited, improvements in AD pathology are observed

The relationship between NETs, which have significant effects on cognition, and AD, which appears to be cognitively impaired, was investigated in a phase 2 study on determining the therapeutic effects against NET dysfunction. In the study, the neuroprotective effect of the NET inhibitor atomoxetine was investigated. As a result of the study, atomoxetine was associated with a significant reduction in CSF tau levels compared to placebo [146]. Considering the current limitation of disease-modifying therapies for AD, the ability of atomoxetine to improve LC function may become useful in clinical practice as an alternative therapeutic solution. Besides targeting NETs, the receptors on which NE exerts strong effects have also been closely studied in AD to find a treatment strategy. In one study, the A1 AR signaling pathway in APP/PS1 mice was inhibited by Terazosin, a clinically tested drug with confirmed tolerability. After A1 AR signal inhibition, improvement in AD pathology and behavioral disorders was observed in the mice [145] (Fig. 3D), suggesting its potential as an AD treatment. The effect of the A1 AR antagonist, Prazosin on memory in an animal model of AD was studied previously, which showed that long-term use of Prazosin may slow the course of AD by increasing astrocytic proliferation, the release of anti-inflammatory cytokines, and the production of APOE [147]. Conversion of Prazosin therapy into active clinical practice in AD patients, especially in the early stages of AD, may be relatively safe as it is currently used in the treatment of hypertension. This may be part of the “drug repurposing” process of Prazosin. The effects of another A1 AR drug, Avenanthramide-C (Avn-C), on LTP and cognition, showed that oral administration of Avn-C to rats induced therapeutic response against AD [148]. Unlike current AD drugs that cause various side effects over long-term usage, Avn-C may not cause these side effects and it is a natural compound found in oats, suggesting greater patient tolerance and compliance to long-term AD treatment. These evidence suggest that NE and its receptors, Ars, may be strong candidates to be targeted for therapeutic strategy against AD, particularly by repurposing current drugs that have already been seen as effective in the noradrenergic system. Another specific physiological role of NE is the ability to regulate microglial functions [154]. As for the case of AD, regulation of inflammation by NE is a significant mechanism, which was shown by an earlier study reporting that NE is able suppress microglia to transcript proinflammatory genes resulting in a decrease of cytokine and chemokine production in N2a APPsw cell line [149]. Another study investigated the link between NE and neuroinflammation by comparing the state of neuroinflammation with degeneration of LC. The study reported a positive correlation with disease status, degeneration of LC-NE neurons, and microglial activation in APP/PS1 mice compared to age match WTs [150] (Fig. 3C). Another study investigated the role of BAR in the NE-mediated suppression by antagonizing β receptors in an AD model of mice. After LPS injection, different β antagonistic (metoprolol, GCP-20712A, atenolol) and agonistic agents (xametorol, mabuterol) were administered at different doses, where the results showed that β blockers worsened inflammation and cognitive function, while the agonists agents attenuated the neuroinflammation. [151]. Finally, as the glia cells express various neurotransmitter receptors and some subtypes of astrocytes release the neurotransmitters (gliotransmitters), it could be possible that NE could indirectly mediate release of other neurotransmitters by regulating the glial activities present in AD. Currently, there is no study directly addressing this question. However, a recent review implicated the astroglial ATP as a slow and steady signaling molecule that is able to regulate neural transmission systems [155]. Additionally, HE is able to trigger ATP release from glia [156]. Finally, NEergic system is affected very early from AD pathology so a mechanism in which neuroinflammation and NEergic system degeneration could lead to a disruption of neural metabolism resulting in CNS wide dysregulation of neurotransmission, ultimately leading to cognitive deficits characteristic of AD. In summary, the Adrenergic system is profoundly altered in AD pathology resulting in severe degeneration of LC, impairments in NE-mediated neurotransmission, pathologic glia inflammation, and severe cognitive dysfunctions.

### Serotonin

Serotonin (5-HT), also known as 5-hydroxytryptamine, is a biogenic amine neurotransmitter. This neurotransmitter functions in the synapses of neurons. 5-HT exerts its effects on cognition, mood, and sleep by binding to neuronal and non-neuronal cell membrane receptors. 5-HT receptors that regulate physiological signaling pathways belong to either G-protein coupled receptors or ligand-gated ion channels. These receptors are divided into seven groups which are 5-HT1 (5-HT1A, 5-HT1B, 5-HT1D, 5-HT1E, and 5-HT1F), 5-HT2 (5-HT2A, 5-HT2B, and 5-HT2C), 5-HT3, 5-HT4, 5-HT5 (5-HT5A, 5-HT5B), 5-HT6, and 5-HT7. The abundance of 5-HT receptors provides a better understanding of the processes involved in serotonergic signaling; however, the lack of suitable selective agents for the receptor subpopulations makes it difficult to reveal their distinctive role, particularly in relation to cognition [157–159].

Serotonin receptors are highly expressed in the nervous system. These receptors are promising therapeutic targets for neuropsychiatric disorders including schizophrenia and depression that may accompany AD as comorbid disorders [160]. Besides the receptors, the changes in level of 5-HT at the synapse in AD patients have also indicated its potential as a therapeutic target [161]. After 5-HT is released into the synaptic region, it is transported back into the pre-synaptic region by 5-HT transporters (SERT), thereby terminating the neuronal signal (122, 123) [162, 163]. Thus, selective serotonin reuptake inhibitors (SSRIs) may have a beneficial role in the pathophysiology of AD [164], ensuring active neuronal signalling is preserved and Aβ and tau fibril deposition are prevented, thereby improving AD. Interestingly, serotonin signaling has been investigated to alter Aβ levels in AD patients and PS1APP transgenic mice with citalopram (5 mg/kg and 10 mg/kg), a type of SSRI [165]. Activation of extracellular regulated kinase (ERK) with serotonin reduces brain Aβ levels in mice. The association between serotonin signaling and Aβ accumulation may be stronger in cognitively challenged individuals. In mouse models, 5-HT degeneration is observed prior to Aβ deposition. In the study conducted as a clinical counterpart, this was investigated molecularly with a partial least squares (mmPLS) algorithm with binary relationship mode. The inter-relationship of Aβ accumulation and low 5-HTT caused cognitive impairment [166] (Fig. 2D). To evaluate neurochemical conditions, mmPLS application can be used in the preclinical period in AD.

The wide variety of 5-HT receptors create various treatment target pathways using antagonistic action. In a study, NAD-229 antagonist for the 5-HT1A receptor and TCB-2 antagonists for the 5-HT2A receptor were able to prevent the progression of AD by reducing neuronal loss and oxidative stress in an AD model (Wistar rats) induced with streptozotocin [167] (Fig. 2D). Its role in learning and memory and reduction of Aβ accumulation highlights the 5-HT4 receptor as a suitable therapeutic target as well. This study further revealed that the upregulation of the 5-HT4 receptor could be observed even in the early phase of AD [168] (Fig. 2D).

In a study, it was found that idalopirdine, an antagonist of 5-HT6 receptors, also has an impact on inhibiting butyrylcholinesterase (BuChE). Obtaining the in vitro BuChE inhibitor was achieved by preparing the carbamate analogue with the presence of benzylaminephenoxide in the structure of idalopyrdine [169], although the findings warrant further investigation. In another study, the effect of idalopyrdine on cognitive performance was investigated in a randomized, double-blind, and placebo-controlled manner for a phase 2 trial, which included 278 patients that received either 90mg of idalopyrdine daily or 10mg of donepezil daily. Idalopirdine was found to improve cognitive function in most patients [170]. Since the 5-HT7 receptor subtype is associated with neurogenesis and hippocampal neuronal function, its roles in apoptosis and long-term potentiation in AD was also investigated. It was found that 5-HT7 receptor activation could improve synaptic dysfunction in AD by reducing apoptosis in the hippocampus [171] (Fig. 2D), but this was more related in treating psychotic symptoms of AD rather than cognition [172]. Thus, 5-HT6 and 5-HT7 receptors may be viewed as a potential target to prevent the progression of AD-related cognitive and psychotic disorders, respectively. Hyperactivity of pyramidal neurons in the CA1 region is an early finding in AD. Abnormal serotonin signals may contribute to increased neural activity in the CA1 region of hAPP-J20 mice. In a study investigating this, researchers used 5-HT1aR and/or 5-HT3aR antagonists and applied whole-cell current-clamp techniques. They found that disrupted 5-HT/5-HT3aR and/or 5-HT/5-HT1aR signaling resulted in heightened excitability of pyramidal neurons and depressed serotonin signaling in the hippocampus of hAPP-J20 mice [173] (Fig. 2D). This irregular signaling could lead to cognitive impairment due to increased neural activity in CA1 pyramidal neurons. The study suggests that activating 5-HT/5HTR with an agonist may enhance hippocampal circuit activity, potentially improving cognitive functions in AD.

Although Aβ accumulation is associated with neuronal hyperexcitability, it is unclear whether it is a cause or a consequence. In the study, there was no difference in spontaneous postsynaptic currents and intrinsic excitability in CA1 pyramidal neurons in an aged APPswe/PS1dE9 AD model [174]. Neuronal excitability may not give a consistent result, especially in older AD models.

In a study conducted on SERT as well as 5-HT receptors, the gene polymorphisms of the 5-HT2A and SLC6A4 transporters were investigated in AD. As a result of the study, polymorphisms were found to not have an effect on their own but may be a risk factor for AD [175]. It may be recommended to increase investigations into these polymorphisms and antagonistic action on 5-HT receptors with larger populations.

Moreover, it is important to highlight the connection between brain-derived neurotrophic factor (BDNF) and its receptor, known as tropomyosin-related kinase receptor type B (TRKB). These substances are produced in the cell bodies of both neurons and glial cells and are associated with 5-HT [176]. BDNF plays a crucial role in regulating neuronal development, plasticity, and synaptic transmission [177]. Although BDNF and serotonin are considered two different systems, they are closely related to each other. BDNF, TRKB, and 5-HT are co-expressed in the median and dorsal raphe of the brain [178]. Studies indicate that BDNF supports the development and survival of 5-HT neurons, while 5-HT stimulates BDNF expression through cAMP-response-element [179]. In the context of AD, neuroinflammation and tau phosphorylation lead to decreased BDNF levels [180]. Enhancing BDNF expression has been linked to improved memory and learning abilities in AD [181, 182]. The interplay between 5-HT and BDNF signaling, along with BDNF's role in regulating serotonergic neurons, may contribute to the therapeutic potential in AD. Recent focus on regenerative neurogenesis in AD has shown that BDNF increases hippocampal neurogenesis by influencing the function and structural plasticity of serotonergic neurons [183]. Another study exploring the relationship between Aβ and serotonin suggests that the decrease in 5-HT production supports the generation of neural stem cells (NSCs). Neuron-glia interactions play a role in NSC production through the 5-HT-BDNF-nerve growth factor receptor pathway in zebrafish [184] (136). Although the regenerative neurogenesis approach through serotonin is considered, it should not be forgotten that it is a complex process (Table 5).

**Table 5:** The role of serotonin in AD based on previous literature

Study type	AD model	Drug and dose	Observation	Remarks	References
In vitro, in vivo	NMRI mice	–	Idalopyrdine, which is a BuChE inhibitor, was also involved in 5-HT6R modulation	Polypharmacology and pleiotropic drugs that affect more than one receptor as may be further investigated	[169]
	Wistar rats	NAD-299 (5 μg/1 μl), NAD-299 (5 μg/0.5 μl), TCB-2 (5 μg/1 μl), TCB-2 (5 μg/0.5 μl)	The wide variety of 5-HT receptors can create treatment pathways with antagonists through exemplary receptors. The effects of the receptor antagonists on neuronal count and oxidative stress in the AD model induced with the drug streptozotocin were investigated	NAD-229 and TCB-2 antagonists may prevent the progression of AD by reducing neuronal loss and oxidative stress	[167]
	Zebrafish	–	The decrease in 5-HT production supports NSC production	Neuron-glia interactions are involved in NSC production through the 5-HT-brain-derived neurotrophic factor-nerve growth factor receptor	[184]
In vitro, in vivo, human study	PS1APP transgenic mice, HC (*n* = 186)	Citalopram (5 mg/kg and 10 mg/kg)	Serotonin signaling has been investigated to alter Aβ levels in AD patients and in a transgenic mouse model	The association between serotonin signaling and Aβ accumulation may be less in cognitively healthy individuals	[165]
In vivo	Wistar rats	AS19 (selective 5-HT7 receptor agonist, 1 μg/μL)	Since the 5-HT7 receptor subtype was associated with neurogenesis and hippocampal neuronal function, its roles in apoptosis and long-term potentiation in AD have been investigated. 5-HT7 receptor activation may improve synaptic dysfunction in AD by reducing apoptosis in the hippocampus	5-HT7 receptors may be viewed as a potential target to prevent the progression of AD	[171]
	hAPP-J20 mice	–	Aberrant serotonergic signals may be associated impaired 5-HT/5-HT3aR and/or 5-HT/5-HT1aR signaling with neuronal hyperexcitability in the CA1 region	This abnormal signaling can cause cognitive impairment with hyperexcitability in pyramidal neurons in CA1	[173]
	PPswe/PS1dE9 and wild-type C57BL/6 J mice	–	There was no difference in spontaneous postsynaptic currents and intrinsic excitability in CA1 pyramidal neurons in an aged APPswe/PS1dE9 AD model	Neuronal excitability may not give a consistent result, especially in older AD models	[174]
Human study	AD patients (*n* = 11), HC (*n* = 12)	–	Its role in learning and memory and reduction of Aβ accumulation highlighted the 5-HT4 receptor as a therapeutic target. The upregulation of the 5-HT4 receptor in the early phase of AD	This may be a compensatory effect to prevent Aβ accumulation and improve cognitive function	[168]
	● AD patients (*n* = 48)	Donepezil (10 mg per day), Idalopirdine (90 mg per day)	It was found that idalopyrdine and donepezil improved cognitive function in LADDER patients	Studies may be conducted on a larger population to confirm this effect of drugs	[170]
	AD patients (*n* = 42), HC (*n* = 18)	–	Although 5-HT7 receptors were associated with psychotic symptoms in AD, it has been found not to be associated with cognitive status	May form antagonists on 5-HT7 receptors for the treatment of psychotic symptoms in AD	[172]
	AD patients (*n* = 252)	–	Polymorphisms do not have an effect on their own but may be a risk factor for AD with a potential combination	It may be recommended to increase the analysis studies that deal with larger populations	[175]
	Mild Cognitive Impairment (*n* = 22), HC (*n* = 27)	–	The interrelationship of Aβ accumulation and low 5-HTT caused cognitive impairment	To evaluate neurochemical conditions, mmPLS application can be used in the preclinical period in AD	[166]

*AD* Alzheimer’s disease, *Aβ* amyloid beta, *HC* healthy controls, *BuChE* butyrylcholinesterase, *LADDER* moderate Alzheimer’s disease, *NSC* neural stem cell, *5-HT* serotonin;5-hydroxytryptamine

### Histamine

Histamine is a neurotransmitter that has been involved in the release of GABA and has a sedative effect on the circadian phases by reducing neuronal activity [185]. Histamine, which is active through the H1, H2, H3, and H4 receptors, acts by regulating different physiological and pathological processes, including immune and pain responses. Neurotransmitters mediated by these four histamine receptors, especially H3 and H4 receptors, have important effects on neuropathic pain modulation [186]. The formation of pathological differences in the neuronal histaminergic system is known to be a major determinant in the formation of cognitive defects. In recent years, acetylcholinesterase (AChE) and H3Rs have been targeted for the treatment of advanced AD [187], suggesting a role of the histaminergic system in AD.

The use of histamine-related agents against triggering brain atrophy is considered neurogenesis-stimulating therapy [188], and may exert its neurogenesis ability in AD condition as well. Histamine in the brain modulates recognition memory and has an effect on major cognitive impairment states. It is thought that the histaminergic systems may have a healing effect on AD and various other neurocognitive disorders [189].

H3Rs expressed in the tuberomamillary nucleus (TMN) of the hypothalamus and the prefrontal cortex (PFC) were examined. In the study, systemic administration of the selective H(3)-agonist, immepip, decreased the firing activity of histamine neurons in TMN and increased the reverse H(3)/H(4)-agonist, thioperamide [190]. Accordingly, it can be concluded that H3Rs, and especially those expressed in the PFC, play an important role in the autoregulation of histamine neurotransmission. As a result, H(3) receptors could be shown as potential targets for future CNS drugs (Fig. 2B). The effect of histamine on hippocampal neuroinflammation has also been evaluated previously. Mice, which were injected intraperitoneally with lipopolysaccharide (LPS) and intrahippocampal histamine, showed that the histamine had reversed glial reactivity and limited impairments in neurogenesis, suggesting histamine exerts an inhibitory effect against glial activation and the release of proinflammatory molecules. It appears that intrahippocampal histamine injection alone induces glial reactivity and causes mild long-term impairments in neurogenesis, reducing the dendritic volume and complexity of immature neurons. Additionally, histamine prevents LPS-induced loss of immature neuron complexity by both CREB and PSD-95 proteins (necessary for proper neuronal activity). Accordingly, it highlights that histamine is a potential therapeutic agent in the treatment of neurological conditions associated with hippocampal neuroinflammation and neurodegeneration [191]. Thus, histamine may be used effectively in the treatment of conditions associated with hippocampal neuroinflammation and neurodegeneration. In order to understand the interactions between glutamatergic and histaminergic systems in the brain, histamine release was investigated in the medial prefrontal cortex and posterior hypothalamus-tuberomamillar nucleus (PH-TMN) using electrophysiological recordings. According to the study, the presence of two subpopulations of NMDA receptors was identified in histaminergic cell bodies in PH-TMN and the latter in GABA-ergic neurons projecting into PH-TMN [192]. In conclusion, it can be said that the histaminergic system is closely regulated by glutamate neurons in various ways.

Besides the neurotransmitter itself, the histamine receptors may also play a role in improving cognition in AD. H1 receptors, which are found on neurons and astrocytes, play a role in recognition memory [185] and in the regulation of anxiety [193]. Similarly, H3 receptors may be targeted, such as with Compound 23, to improve memory [194]. Unlike H1 receptor, H3 receptors has a therapeutic effect through antagonism activity. DL77, an H3 receptor antagonist, was also found to have an ameliorating effect on memory deficits caused by MK801 [195]. Histamine, a brain monoamine, plays an important role in the treatment of cognition. According to the study, thioperamide, an inverse agonist of Histamine-3 receptors, was found to increase the firing activity of dopamine neurons in the ventral tegmental area [196]. In conclusion, antagonists of Histamine-3 receptors may be useful in the treatment of cognitive loss because of their potential to stimulate monoamine neurotransmission (Fig. 2A).

Considering this, acute systemic injection of DL77 may have memory-enhancing effects for AD. Similarly, E177, also an antagonist of H3 receptor, was found to have similar ameliorating effect on memory disorders caused by acute pentylenetetrazol [197]. Thus, H3 receptors may be used as a potential target for diseases, such as AD that causes memory disorders. Histamine also stimulates the production of nitric oxide (NO) from human cerebrovascular endothelial cells, where it has an effect on calcium ion (Ca^2+^) release in the cell and oxide release in the endothelial brain microvascular circulation (Fig. 2B). Accordingly, histamine induces Ca^2+^ release in the cell triggered by H1 receptors by promoting Ca^2+^ release via InsP3R3 and TPC1-2 [198]. As a result, H1 receptor antagonism may also be seen as therapeutic candidates for AD (Table 6).

**Table 6:** The role of histamine in AD based on previous literature

Study type	AD model	Drug and dose	Observation	Remarks	References
In vitro	hCMEC/D3 cells were loaded with the Ca^2+^	Fura-2/AM (1 Mm)	Histamine induced Ca^2+^ release in the cell triggered by H1Rs by supporting Ca^2+^ release via InsP3R3 and TPC1-2	Investigation of the role of increased Ca^2+^ concentration in H1Rs in the detection of disease may shed light on the treatment of AD	[198]
In vivo In vitro, in vivo, Human study	MK801 amnesia rats	(IC50 ¼ 880 nM and 394 nM) and hMAO B (IC50 ¼ 775 nM)	Compound 23 has a memory-enhancing effect in dizocilpine-induced amnesia in rats	The favorable analgesic effects of compound 23 in neuropathic pain models can be derived from the design of new MTDLs	[194]
In vivo	Animal model of Hrh1 cKO mice	_	H1R has a regulatory effect on increased aggressive behavior, altered circadian rhythm, and quality of alertness	The effects of H1R on cognitive memory may be used in plasticity-based pharmacological studies	[193]
	Male Wistar rats	Immepip (0.01–0.15 mg/kg), thioperamide (0.05–0.50 mg/kg)	Immepip has been found to decrease the firing activity of histamine neurons in TMN and increase the reverse H(3)/H(4)-agonist, thioperamide	H(3) receptors could be cited as potential targets for future CNS drugs	[190]
	Rats (*n* = 11)	mGluR 2 and 3	Histamine may be used in the treatment of conditions associated with hippocampal neuroinflammation and neurodegeneration	NMDA receptors was identified in histaminergic cell bodies in PH-TMN and the latter in GABA-ergic neurons projecting into PH-TMN	[192]
	MK801 adult male rats	DL77 (2.5, 5, and 10 mg/kg), RAMH (10 mg/kg)	Pro-cognitive effects shown in NOR and PAP tests were not associated with natural movements and emotional changes	Paradigms of acute systemic injection of DL77 may have memory-enhancing effects	[195]
	Animal model of Inbred male rats weighing between 180 and 220 g in AD	PTZ (60 mg/kg)	E177 has a therapeutic effect on memory disorders caused by acute PTZ	H3Rs may be used as an important target for diseases that cause memory disorders	[197]
	C57BL/6 J male mice	LPS (2 mg/kg)	Histamine prevented LPS-induced CREB and PSD-95 protein loss and immature neuron complexity	Histamine may be used in the treatment of neurological conditions associated with hippocampal neurodegeneration	[191]

*MCI* mild cognitive impairment, *APP* amyloid-beta precursor protein, *LPS* lipopolysaccharides, *Hrh1 Cko* H1 receptor gene conditional knockout, *IC50* acetylcholinesterase, *hMAO* human MAO B, *H1R* histamine 1 receptor, *MTDLs* multitarget-directed ligands, *PAP* passive avoidance paradigm, *NOR* novel object paradigm, *PTZ* pentylenetetrazole, *Compound 23* 2-(5-(azepan-1-yl) pentyloxy)-9H-xanthen-9-one, *MK801* dizocilpine, *TMN* tuberomammillary nucleus, *mGluR 2 and 3* metabotropic glutamate receptors, *PH-TMN* posterior hypothalamus-tuberomamillary nucleus

## Alkaloid-based Neurotransmitters

### Acetylcholine

Acetylcholine (ACh), composed of acetic acid and a choline ester, is a chemical messenger in the cholinergic system that works as a neurotransmitter between the autonomic nervous system and the neuromuscular system [199]. ACh receptors are divided into muscarinic and nicotinic receptors. Muscarinic ACh receptors (mAChR) belong to the class I (rhodopsin-like) G protein-coupled receptor family. Expressional and functional changes on mAChRs are associated with the pathogenesis of neurological diseases such as AD, and may be targets for intervention [200, 201]. On the other hand, nicotinic ACh receptors (nAChR), which provide rapid neurotransmission in the central and peripheral nervous system, belong to ligand-gated ion channels and have various subunits that can combine to form different nAChR subtypes [202]. These receptors consist of homomeric α subunits or heteromeric α and β subunits [203]. Different nAChR oligomers, whose roles vary depending on their anatomical location in cholinergic circuits, are expressed especially in memory-related brain regions including hippocampus, frontal cortex, substantia nigra, and thalamus [204, 205]. A study aimed at elucidating the role of nAChRs in memory revealed that α7 nAChRs contribute to long-term strengthening, while α4β2-containing nAChRs are effective in the recovery of associative memory [206]. Additionally, nAChR is effective in the activation of inhibitory interneurons in the CA1 region. Optogenetic release of ACh was performed and its effect on pyramidal and different interneurons was measured with patch-clamp. Activation of α4β2* nAChR in interneuron-selective interneurons has been shown to promote further inhibition [207]. Interneurons innervating pyramidal neurons may be less affected.

ACh is transported into synaptic vesicles by the high-affinity choline uptake transporter (CHT) and the vesicular ACh transporter (VAChT). Defects in regulating the amount of ACh in the presynaptic and postsynaptic regions constitute a risk factor for AD [208]. The cholinergic system, which consists of the ACh neurotransmitter, transporter, and receptors, is one of the most crucial regulatory neurotransmitter systems for learning and memory and selective attention-related behaviors.

In examining the correlation between AD and the cholinergic system, the hypothesis of auto-cannibalism emerges. According to this hypothesis, cholinergic neurons try to obtain free choline by hydrolyzing membrane phospholipids in choline deficiency. This situation causes abnormal proteolysis of the β-amyloid precursor protein and the release of enzymes that lead to the formation of the β-A4 amyloid protein, making cholinergic neurons more susceptible to injury [209–211]. According to the cholinergic hypothesis, the decrease in cholinergic neurotransmission triggers the decline in cognitive functions that occur in AD, such that drugs modulating the cholinergic system are able to alleviate a number of cognitive and non-cognitive symptoms [212, 213]. To support this, a mouse model of AD showed that the response of extra-telencephalic projection (ET) neurons in the prefrontal cortex (PFC), which is responsible for short-term working memory to familiar objects, decreased due to ACh deficiency, thereby impacting recognition memory [214]. In another recent study, the impact of ACh deficiency on neural oscillation was witnessed through the EEG slowdown that was recorded in the AD model [215]. Thus, the cholinergic system may be targeted for the diagnosis and treatment of AD.

Sleep-disordered breathing (SDB) in AD triggers cholinergic basal forebrain degeneration as well as increases Aβ level. Blood oxygen levels should be restored to prevent the pathological changes caused by SDB, which subsequently may impair cognitive function in AD [216]. Another factor triggering cognitive decline in AD is the disruption of M1 (muscarinic) receptor activity. M1 receptors, the most prevalent among muscarinic receptors, are distributed throughout crucial areas of the forebrain, including the hippocampus, neostriatum, cerebral cortex, and thalamus [217, 218]. At the cellular level, they predominantly reside post-synaptically and are abundant in diverse cell types, such as striatonigral pyramidal excitatory neurons, inhibitory GABAergic neurons, and glia [219, 220]. The expression of M1 receptors spans various brain regions, enabling them to play a pivotal role in a range of physiological and pathological functions, including attention, synaptic plasticity, learning, and memory [221, 222]. Evidence suggests that deleting M1 receptors in 3xTgAD and transgenic mice models or reducing their activity in AD patients can lead to an elevated astrocytic and microglial response associated with Aβ plaques [223] (Fig. 1C). The deletion of the M1 receptor resulted in enhanced astrogliosis and microgliosis, inducing the deposition of fibrillar Aβ and tau hyperphosphorylation. Additionally, it led to an increase in interleukin-1β and tumor necrosis factor-α. These situations demonstrate the role of the M1 receptor in neuroinflammation. In AD, glutamatergic deficiency and loss of neuronal protein kinase C (PKC) signaling, both involved in cognitive processes, are associated with impaired M1 receptor signaling as well [224]. Modulation of the M1 receptor has been studied in a Tg2576 transgenic AD mouse model (APP_695_SWE) with donepezil (0.1, 0.3, 1, and 3 mg/kg) and PQCA (0.1, 1, and 10 mg/kg), where selective positive allosteric M1 receptor modulation reduced learning and memory deficits [225]. In the early stages, alterations in the cholinergic system can be identified by utilizing mouse models featuring genetically mutated APP genes, like TG2576, PDAPP, APP/PS1, and APP23, which lead to increased production of Aβ. Alternatively, acute changes can be observed by administering Aβ(1–42) through intracerebroventricular or intrahippocampal injections [226, 227]. These rapid alterations result in toxicity to cholinergic cells, leading to impaired acetylcholine (ACh) release and synthesis in neurotransmission processes [228].

In addition to mAChR receptors, nAChR receptors may also be involved in cognitive dysfunction in AD. The α4β2 subtype of nAChRs was found severely decreased in AD; however, it was not associated with the cognitive disorders observed in the early period [229]. Cognitive function in AD may be more related to α7nAChRs. The inadequacy of anti-Aβ approaches used to treat AD has been demonstrated by the deletion of α7nAChR on α7 knock-out mice [230]. The α7nAChR-mediated therapeutic approaches that restore Aβ function may be considered prior to Aβ and tau pathology. Aβ affects α7 function as an agonist and a negative regulator, where low Aβ density was beneficial for α7 activation, and high Aβ density decreased α7 activity [231]. Thus, exposure to high levels of Aβ may initiate and progress AD by causing a signal deficiency in the cholinergic system. The association of α7-nAChR with learning and memory increases research on this receptor subtype. There is also a subtype of nAChR in which α7 subunits are expressed together with β2 subunits in the study, which was carried out by considering basal forebrain cholinergic neurons that are sensitive to Aβ. With inhibition of Aβ1-42, whole-cell current-clamp results on α7β2-nAChRs showed slower currents and higher sensitivity [232]. α7β2-nAChRs may be a possible treatment option for changes in cholinergic signaling.

Acetylcholinesterase/cholinesterase (AChE/ChEI) inhibitors have been the main drugs used for the treatment of AD. These drugs enhance cholinergic neurotransmission and reduce Aβ accumulation by preventing the hydrolysis of ACh [233]. While tacrine was the first approved drug for AD, it is no longer in use due to its harmful effects on the liver. Currently, commercially available drugs for AD include donepezil, galantamine, and rivastigmine. Donepezil and galantamine are reversible inhibitors of AChE, while rivastigmine is a pseudo-reversible inhibitor of both AChE and BuChE [234–236]. The initial dose of donepezil typically ranges from 5 to 10 mg per day, but it can be increased to 23 mg per day for moderate to severe cases, despite potential side effects [237] . For mild to moderate AD patients, rivastigmine is administered at a dosage of 3–12 mg per day, and it is available in transdermal patches, oral capsules, and liquid form [238]. Additionally, for these patients, galantamine is given at a dosage of 16–24 mg per day [239]. NMDA receptor antagonists, antioxidant agents, and AChE combination therapy may also be used against AD [240]. Since downregulation of the cholinergic neurotransmission increases/exacerbates the deterioration in cognitive functions in AD, increasing ACh levels may be a promising therapeutic approach to ameliorate AD (Table 7).

**Table 7:** The role of acetylcholine in AD based on previous literature

Study type	AD model	Drug and dose	Observation	Remarks	References
In vitro	BOSC-23 cells, derived from HEK 293 cells	ACh and human amyloid-β1–42	Aβ affected α7 function as an agonist and a negative regulator, while low Aβ density was beneficial for α7 activation, and high Aβ density decreased α7 activity	Exposure to Aβ may initiate and progress AD by causing a signal deficiency in the cholinergic system	[231]
	C57BL/6 or nAChR 2 knock-out mice on a C57BL/6 neurons	–	With inhibition of Aβ1-42, whole-cell current-clamp results on α7β2-nAChRs showed slower currents and higher sensitivity	α7β2-nAChRs may be a possible treatment option for changes in cholinergic signaling	[232]
In vitro, in vivo	3xTgAD and transgenic mice	–	Deletion of M1 receptors in mouse models or decreased activity in AD patients increased the astrocytic and microglial response associated with Aβ plaques	Increasing the use of M1 receptor agonists for the treatment of AD may be considered	[223]
	α7 knock-out mice	α-Bungarotoxin (10 μM), methyllycaconitine (10 μM), murine anti-Aβ antibody M3.2 (2 μg/mL)	The inadequacy of anti-Aβ approaches used to treat AD has been demonstrated by deletion of the α7nAChR	The α7nAChR-mediated therapeutic approaches that restore Aβ function may be considered prior to Aβ and tau pathology	[230]
In vivo	Heterozygous 5 × FAD mice line of C57B/6 J	–	ACh deficiency in a mouse model of AD showed that the response of ET neurons in the PFC responsible for short-term working memory to familiar objects decreased due to ACh deficiency	Impairments in ORM in mice may be revealed by a neural mechanism related to the cholinergic system	[214]
	Mice model of C57BL/6, APP/PS1	–	Blood oxygen levels should be restored to prevent the pathological changes caused by SDB, which impaired cognitive function in AD	SDB may contribute to the pathological process of AD by inducing cholinergic basal forebrain degeneration	[216]
	Tg2576 transgenic mice	Donepezil (0.1, 0.3, 1, and 3 mg/kg), PQCA (0.1, 1, and 10 mg/kg)	Selective positive allosteric M1 receptor modulator PQCA was observed to reduce learning and memory deficits in AD model mice	M1 positive allosteric modulators including PQCA may offer a therapeutic approach for AD treatment	[225]
	Mice	–	Activation of α4β2* nAChR in interneuron-selective interneurons has been shown to promote further inhibition	Interneurons innervating pyramidal neurons may be less affected	[207]
Human study	AD patients (*n* = 22), HC (*n* = 12)	–	The resulting cholinergic dysfunction, PKC signaling, and NMDA receptors have been found to be co-factors of impaired cognitive characteristics in AD	The cholinergic system may be a promising candidate for the treatment of cognitive disorders in AD	[224]
	AD (*n* = 15), HC (*n* = 14)	–	The relationship between nAChR and cognitive function in AD indicated that α4β2 subtype of nAChRs were severely decreased in AD and may not be associated with cognitive disorders observed in the early period	Cognitive function in AD may be more related to α7nAChRs and the association with selective ligands can be examined	[229]

*ACh* acetylcholine, *AD* Alzheimer’s disease, *EEG* electroencephalogram, *ET* extratelencephalic projection, *HC* healthy controls, *M1 receptor* muscarinic acetylcholine receptor M1, *NMDAR* N-methyl-d-aspartate receptor, *ORM* object-relational mapping, *PFC* prefrontal cortex, *PKC* protein kinase C, *SDB* sleep-disordered breathing, *α7nAChR* alpha-7 nicotinic receptor

## Ethanamide-structural Neurotransmitters

### Melatonin

Melatonin or 5 methoxy-N-acetyltryptamine is a neurohormone primarily secreted in the pineal gland [241]. Melatonin is critical in the mediation of day-night system cycles in the body, including sleep [242]. Melatonin-dependent physiological and psychological regulation is thought to be a mechanism (circadian rhythm) to adapt to changing external environmental conditions in regular cycles [242–244]. In the pinealocytes (pineal gland), after the conversion of the precursor molecule l-tryptophan to serotonin, melatonin is synthesized as a result of acetylation and methylation of serotonin [245].

In addition to the regulation of the biological clock, melatonin also contributes to mitochondrial processes including binding of free radicals, thereby protecting against oxidative stress [246]. This suggest there may be a protective effect of melatonin against neurological diseases as well [247–249]. MT1 and MT2 are G-protein high-affinity receptors for melatonin [250] (179). MT1 and MT2 are involved in peripheral and central biological clocks, so they appear to be widely spread throughout the body [250] (Fig. 2C).

Sleep disorders, major depressive disorders, and the pharmacological mechanism of many drugs with neuroprotective effects have included targeting MT1 and MT2 receptors [251]. Due to melatonin’s strong anti-oxidative property, free radical scavenging capacity, and neuroprotective ability, the relationship between melatonin and AD should be explored further. Melatonin levels in the cerebrospinal fluid (CSF) have been observed to be significantly reduced in age-matched AD individuals compared to control groups [12, 252]. Similarly, studies have also found that serum melatonin levels tend to decrease in AD [12, 253]. A thorough understanding of how serum melatonin is affected in AD may be important in monitoring the disease progression with relatively low-invasive procedures. The decrease in melatonin levels may be the source of the sleep disturbances in AD. One of the potential causes of low melatonin levels in AD may be epiphyseal calcification [254]. The pineal glands of individuals with AD were examined by computed tomography (CT), where significantly increased calcified gland volume was reported in individuals with AD compared to the control group [255]. This may also be a valuable marker for early detection of AD. Besides melatonin levels, alteration in MT1 receptors in the brain may also contribute to AD. One study reported that the MT1 receptor in the suprachiasmatic nucleus (SCN) was significantly reduced in elderly and AD individuals [255].

Sleep deprivation is an important risk factor for AD [256]. Considering that melatonin, which undergoes various changes in AD, provides the main input for circadian clock, there may be a bidirectional interaction between AD and circadian rhythm disorders. The effect of chronic sleep deprivation was studied in mice with AD, where abnormalities in the expression of clock genes were reported [257]. Expression of key clock genes including CLOCK and BMAL1 which may be regulated by melatonin may induce potent anti-oxidative responses [258, 259] that could lead to AD, among other neurological disorders.

The effect of melatonin-inducible SIRT1 gene expression on Aβ accumulation and oxidative stress have also been investigated using a SAMP8 animal model. In this study, induction of melatonin-dependent SIRT1 increased the survival of SAMP8 neurons [260], which may have reduced Aβ accumulation and oxidative stress, thus indicating that the SIRT1 pathway may be a possible target for the treatment of AD. Besides that, in a neuronal culture study, melatonin provided a positive regulation of ADAM10 and ADAM17 transcription factors and increased ADAM10 and ADAM17 expression, which increased non-amyloidogenic APP processing [261]. Additionally, melatonin may play a protective but non-therapeutic role against AD by preserving its α-secretase activity. Melatonin may also modulate the endoplasmic reticulum (ER) stress-related pathways, thereby exhibiting an ameliorating effect on tau hyperphosphorylation [262]. In support, intraperitoneal injection of melatonin in a mice model had a reducing effect on tau hyperphosphorylation and an improvement in memory function, which was accompanied by a decrease in Aβ accumulation [263]. The effect of melatonin on tau phosphorylation and APP may indicate that melatonin may be more than a protective agent for AD (Table 8). Another pathway for the melatonin-related AD amelioration could be Notch1 signaling. In a study done on the cerebral cortex of rats, melatonin administration restored deregulated levels of Notch1, NICD, and HES1 [264]. A better understanding of the relationship between Notch1 axis and melatonin may yield a valuable way of treating AD (Fig. 2C).

**Table 8:** The role of melatonin in AD based on previous literature

Study type	AD model	Drug and dose	Observation	Remarks	References
Human study	Post-mortem human brain tissue	–	Significant decrease in the CSF melatonin level was found in the age-matched AD individuals compared to the control groups	Decreased melatonin levels are likely to be the source of the sleep disturbances seen in AD	[252]
	Clinical trial	–	It has been determined that serum melatonin levels tend to decrease in AD	Serum melatonin affected in AD may be an important step in monitoring the disease with relatively low-invasive procedures	[253]
	Post-mortem human brain tissue	–	Significantly increased calcified pineal gland volume was detected in individuals with AD compared to the control group	Monitoring pineal gland integrity, serum, and CSF melatonin levels may play a role in early detection of AD	[255]
In vivo	APP/PS1	–	CSD aggravated AD pathology and caused abnormalities in the expression of clock genes	CSD may be a factor rather than a result of AD	[257]
	Sprague–Dawley	Melatonin (50 mg/kg)	AB1-42 oligomers reduced the expression of Notch1, NICD, and Hes1, three major members of the Notch1 signaling pathway	For Alzheimer's disease and other age-related neurodegenerative illnesses, Notch1 signaling may offer a viable treatment option	[264]
	C56BL/6N	Melatonin (10 mg/kg)	Intraperitoneal melatonin reduced tau hyperphosphorylation	Intraperitoneal injection of melatonin showed a reducing effect on tau hyperphosphorylation	[263]
In vitro	SAMP8 neuron culture	Melatonin (1 mM), restravol (50 μM)	Melatonin-dependent induction of SIRT1 increased the survival of SAMP8 neurons	SIRT1 pathway may be a possible target for AD treatment	[260]
	HumanHEK293 cells, mouse embryonic fibroblasts	–	Melatonin decreased Aβ plaque formation by providing positive regulation of ADAM10 and ADAM17 transcription factors	Melatonin in early AD may play a protective but non-therapeutic role against AD by preserving α-secretase activity	[261]
	Sprague- Dawley hippocampal Neuron Culture	Melatonin (50 μM)	Melatonin-induced ER stress pathway modification has been found to improve tau hyperphosphorylation	It may be possible to say that one of the mechanisms underlying the protective effect of melatonin is the regulation of ER stress	[262]

*AD* Alzheimer’s disease, *CSD* chronic sleep deprivation, *SCN* suprachiasmatic nucleus, *CSF* cerebrospinal fluid, *MT* melatonin receptor, *ER* endoplasmic reticulum, *AB* amyloid beta

## Gas-based Neurotransmitters

### Nitric Oxide

Nitric oxide (NO) is a member of gaseous signal molecules (gasotransmitter) which plays a role in intracellular signaling of neurons [265]. NO can passively penetrate the cell membrane and change its intracellular target, a feature that differs from other neurotransmitters [266, 267]. NO is synthesized from the amino acid l-arginine by NO synthase (NOS) [268]. NOS exhibits three isoforms, which are endothelial NOS (eNOS), neuronal NOS (nNOS), and inducible NOS (iNOS) [269]. Among these isoforms, the function of nNOS and eNOS depends on calcium and calmodulin. However, iNOS is independent of calcium and calmodulin [270]. The half life of NO produced by NOS is short due to their gaseous form. Physiological role of NO in the tissue depends on the concentration [271]. The interaction between NO and superoxide radicals can lead to the formation of peroxynitrite. Peroxynitrite, a strong inducer of cell death, can lead to inflammation, vascular diseases, and neurodegeneration [272].

Soluble guanylate cyclase (sGC) is the intracellular enzyme receptor of the NO molecule. NO stimulates sGC and converts guanosine triphosphate (GTP) to the second messenger, cyclic guanosine monophosphate (cGMP) [273]. Thus, the sGC enzyme plays a major role in NO/cGMP signaling [274] which is important for cell signaling and learning and memory [275]. NO/cGMP signaling also induces cAMP response element binding (CREB) phosphorylation, which is involved in the synthesis of plasticity-related proteins. NO/cGMP/CREB signaling decreases with age and in neurodegenerative diseases. It may also be affected by tau protein and Aβ peptide of AD [276]. Due to this, the level and distribution of NO may be of vital importance for AD development and progression.

The effect of partial endothelial NO synthase (eNOS) deficiency on cognitive deficit and amyloid pathology was investigated in a mouse model, where partial eNOS deficiency was found to increase behavioral dysfunction, Aβ accumulation, and microglial pathology in APP/PS1 mice, leading to the pathogenesis of AD [277] (Fig. 3A). In a rat model of AD, NO in the dentate gyrus impaired the glutamate response during spatial learning [278]. This suggests that NO may impair spatial learning, memory, and related synaptic plasticity in AD rats. The effect of protein arginine methyltransferase 4 (PRMT4) enzyme on NOS and cerebral blood flow in AD was investigated. Accordingly, inhibition of PRMT4 increases NO production in 3xTg-AD mice [279]. PRMT4 signaling may have an important role in the pathogenesis of AD-associated cerebrovascular dysregulation. The effect of the Tau binding protein nNOS on the carboxy-terminal PDZ ligand (CAPON) on tau aggregation and neurodegeneration was investigated on the AppNL-G-F mouse model. Accordingly, overexpression of the CAPON protein in the hippocampus leads to neurodegeneration by increasing the death of neuron cells [280]. CAPON may be a new treatment option for AD treatments.

The role of NO signaling in naringin protection against intranasal manganese and intracerebroventricular Aβ-induced neurotoxicity was seen in rats, where NO maintained protection against intranasal manganese-induced memory impairment, through its antioxidant, anti-inflammatory, and anti-amyloidogenesis properties [281]. In another study with rats, the effect of NO-like chemical on neurobehavioral and biochemical changes in a rat model of AD showed that the NO releaser, s-nitrosoglutathione (GSNO) (1 ml/kg), ameliorates cognitive deficits and associated brain biochemical changes in an AD model [282]. Accordingly, the interactions of NO and BDNF may contribute to this effect. In the study using electrophysiological techniques, the relationship between the synaptic and memory dysfunction induced by Tau oligomers and the NO cascade was investigated. Accordingly, the findings highlight the importance of upregulation in the NO cascade, the second messenger pathway that can be used to counter Tau-induced damage to synaptic plasticity and memory [283]. The NO cascade may offer new therapeutic approaches against diseases characterized by an increase in Tau oligomers, such as AD and other neurodegenerative diseases. In an experimental study, the effect of sulforapne (25 mg/kg and 50 mg/kg), which reduces NO release, on neuroinflammation and hyperphosphorylated Tau protein through regulation of the PI3K/Akt/GSK-3 β pathway in experimental models of AD was investigated. Accordingly, sulforaphane exerts a protective effect against cognitive deficits and neuroinflammation through modulation of the PI3K/Akt/GSK-3β pathway and inhibition of NF-kB activation [284]. Sulforaphane is a promising neuroprotective agent that needs further investigation in the treatment of AD. The effect of Aβ plaques on NO producing neurons in a transgenic mouse model of AD had induced Aβ plaques for neuritic dystrophy in cortical neurons containing NOS [285]. This induction may be related to the mechanism of Aβ neurotoxicity in human AD.

NO signaling is used as a compensatory mechanism to maintain synaptic plasticity in AD mice. Therefore, NO uptake increases synaptic transmission and plasticity in early AD stages [286] (Fig. 3B). Although this has a positive effect at first, the cumulative effects of NO and S-nitrosylation may cause greater damage over time. In this way, this accumulation may accelerate AD pathology. NOS activity in the brain microvessels of AD patients showed a significant increase [287]. Accordingly, the high production of NO, the neurotoxic mediator, in the CNS may have an impact on the susceptibility of neurons to injury and cell death in AD (Table 9).

**Table 9:** The role of nitric oxide in AD based on previous literature

Study type	AD model	Drug and dose	Observation	Remarks	References
In vivo	Sprague–Dawley rats	L-NMMA (1 μg/μL)	NO in the dentate gyrus impaired the glutamate response during spatial learning	NO may impair spatial learning, memory, and related synaptic plasticity in AD rats	[278]
	Wistar rats (*n* = 32)	STZ (3 mg/kg), GSNO (1 ml/kg), LA HCl (1 ml/kg)	GSNO, a NO releaser, ameliorated cognitive deficits, and associated brain biochemical changes in a slow-developing AD model	NO and BDNF interactions may play an essential role	[282]
In vitro, in vivo	3xTg‐AD	TP‐064 (30 mg/kg)	The effect of PRMT4 on NOS and cerebral blood flow in AD was investigated. Accordingly, inhibition of PRMT4 increases NO production in 3xTg-AD mice	PRMT4 signaling may have an important role in the pathogenesis of AD-associated cerebrovascular dysregulation	[279]
In vitro, in vivo	App^NL−G−F^ mouse, hTau-KI mice	-	Neuronal cell death in the CAPON-expressing hippocampus suggests that CAPON accumulation increases neurodegeneration	CAPON may be a new therapeutic target for AD treatments	[280]
In vitro, in vivo	Sprague–Dawley rats	SF (25 mg/kg and 50 mg/kg), donepezil (5 mg/kg)	SF exerts neuroprotective effects against cognitive deficits and neuroinflammation through modulation of the PI3K/Akt/GSK-3β pathway and inhibition of NF-kB activation	SF is a promising neuroprotective agent that should be investigated further in the treatment of AD	[284]
In vitro, in vivo	C57BL/6 mice	DMSO	Results highlights the importance of upregulation in the NO cascade, the second messenger pathway that can be used to counteract tau-induced damage to synaptic plasticity and memory	The NO cascade may offer a new therapeutic approach against diseases characterized by an increase in Tau oligomers, such as AD and other neurodegenerative diseases	[283]
In vitro, in vivo	APP/PS1 mouse	–	Partial eNOS deficiency increased behavioral dysfunction, Aβ brain accumulation, and microglial pathology in APP/PS1 mice. Also, endothelial dysfunction has a role in the pathogenesis of AD	Current findings may provide the scientific basis for developing preventive and therapeutic strategies for endothelial dysfunction	[277]
	Wistar rats	LA (50 mg/kg), L-NAME (5 mg/kg)	Naringin provided protection against ICV β-A1-42. It also provides protection against intranasal manganese-induced memory impairment	Naringin may have therapeutic potential for AD	[281]
In vitro, in vivo	Transgenic huAPP mice (*n* = 21)	–	Aβ plaques induced neuritic dystrophy in NOS-containing cortical neurons in the AD model	This may be related to the mechanism of Aβ neurotoxicity in human AD	[285]
	3xTg mouse	L-NAME (200 μM)	NO uptake may play a compensatory role to increase synaptic transmission and plasticity in early AD stages	This may have a positive effect at first. However, over time, the cumulative effects of NO and S-nitrosylation may cause major damage and accelerate AD pathology	[286]
Human study	Brains from patients that died from AD (*n* = 9), HC (*n* = 8)	–	There was a significant increase in NOS activity in micro vessels isolated from the brains of AD patients	High production of NO, the neurotoxic mediator, in the CNS may have an impact on the susceptibility of neurons to injury and cell death in AD	[287]

*NO* nitric oxide, *STZ* streptozotocin, *GSNO* S-nitrosoglutathione, *eNOS* endothelial nitric oxide synthase, *Aβ* β-amyloid, AD Alzheimer’s disease, *ICV* intracerebroventricular, *BDNF* brain-derived neurotrophic factor, *PRMT4* protein arginine methyltransferase 4, *NOS* nitric oxide synthase, *CNS* central nervous system, *nNOS* neuronal nitric oxide synthase, *CAPON* carboxy-terminal PDZ ligand of nNOS, *SF* sulforaphene, *DMSO* dimethyl sulfoxide, *LA* l-arginine, *L-NMMA* a nitric oxide synthase inhibitor, *L-NAME* N(G)-nitro-l-arginine methyl ester, *huAPP* human β-amyloid precursor protein, *HC* healthy controls

## Purine-based Neurotransmitter Regulator

### Adenosine

Adenosine is a purine nucleoside [288] located inside the cell or on its surface as a result of the breakdown of adenine nucleotides. Adenosine formation also increases with changing stress levels [289]. Adenosine acts as an upstream regulator of a wide range of neurotransmitters, receptors, and signaling pathways. Adenosine interacts with G protein subgroup receptors A1, A2A, A2B, and A3 receptors [288]. Localization of the receptors varies. In the cortex, hippocampus, and cerebellum, A1R is ubiquitously found. For A2R, they are distributed in the striatum and olfactory bulb. A2BR and A3R are localized in the low levels of expression [290].

Adenosine plays an important role in energy production, cellular metabolism, and gene regulation in brain cells. The continuation of these duties prioritizes the maintenance of intracellular and extracellular adenosine homeostasis. It is observed that adenosine homeostasis may be impaired in pathophysiological conditions [291].

**Fig. 4:**
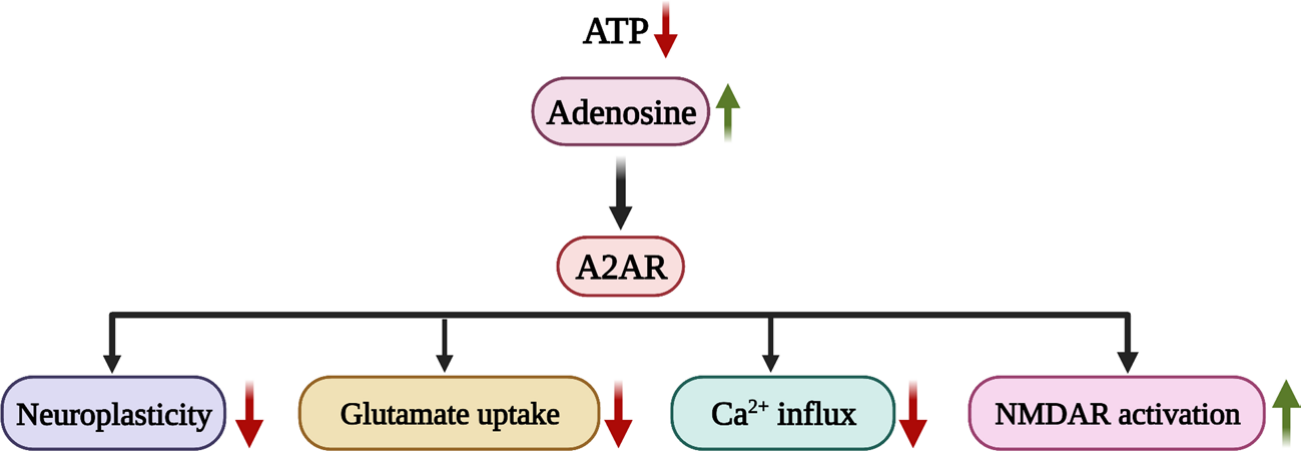
A2AR signaling events in CNS. Adenosine accumulation in the central nervous system (CNS), activates A2A receptors (A2AR). The activated receptor can be associated with neurodegeneration by its effects on neuroplasticity, glutamate uptake, NMDAR activation, and Ca2+ influx. This accumulation is more significant in the hippocampus

Adenosine may play a homeostatic modulator role by activating adenosine receptors, especially A1 and A2A receptors and may also act as a neuromodulator at the synaptic level [292]. It is known that adenosine receptors play a role in various pathophysiological responses, especially in vasodilation, pain, and inflammation. A1 and A3 receptors inhibit adenylyl cyclase, while A2A and A2B receptors stimulate adenylyl cyclase [293]. The roles of adenosine in neuromodulation and neuronal disorders may be similar. This causes A1 receptors to become an obstacle to overcome the onset of neurodegeneration. A1 receptors effectively control neurodegeneration only in certain situations. Unlike A1 receptor, A2A receptor alleviates the burden of neuronal disorders in the long term in neurodegenerative diseases such as AD [294]. A2A can be associated with neurodegeneration. Its activation in the hippocampal region assumes different regulatory functions. Regulations of synaptic plasticity, calcium influx, glutamate release, and NMDA activation are the primary ones among these. In AD patients, A2A expression is increased more in the hippocampus than in the frontal cortex. This difference indicates that A2AR may be a potential biomarker of AD [295] (Fig. 4).

An increase in extracellular adenosine produced from ATP was found in a 2019 study to be associated with synaptic and memory dysfunction in early AD. According to the results, overactivity of A2AR leads to synaptic and memory dysfunction in early AD. In the study, it was observed that ecto-5′-nucleotidase (CD73)-mediated adenosine was effective in the activation of A2AR [296]. Studies targeting CD73 activity may show results in AD treatment by providing A2AR regulation. According to a study conducted in 2021, the relationship between A2AR and Connexin 43 (Cx43) is involved in regulating hemicanal activity and ATP release in Aβ-exposed astrocytes. Findings show that A2AR increases Cx43 activity [297]. It is seen that this relationship can sustain A2AR activity in AD-like conditions.

Upon examining the effect of A2A receptor blockade on early memory and plasticity deficits in a triple transgenic mouse model of AD, a therapeutic-like ability of A2A receptor antagonists was suggested [293]. This therapeutic-like ability may ameliorate synaptic disorders and memory dysfunctions in early AD. Another study also observed the therapeutic effect of A2A receptor antagonists in reducing amyloid pathology in a mouse AD model [298]. These evidences supported the idea that A2A receptor blockade has therapeutic value in AD. Besides that, the A2A receptor activation abolished long-term potentiation at synapses in a APP/PS1 mouse model with AD [299]. The results attained supported the idea that the therapeutic effects of A2A receptor antagonists in early AD patients should be studied.

In another study, caffeine and A2A receptor antagonists were thought to play a role in preventing cognitive deficits caused by Aβ [300]. This study provided the first direct in vivo evidence that caffeine and A2A receptor antagonists protect against Aβ-induced amnesia in AD management by protecting against Aβ neurotoxicity [301] (Fig. 1D). According to a 2020 study, A2AR antagonists such as istradefylline affect NMDA glutamate receptor functions. The study using transgenic mouse neuron and microglia cultures showed that NMDA and A2A receptors could interact and form complexes [302]. The results indicate that A2AR antagonists can provide neuroprotection in AD (Fig. 1D). A study published in 2021 suggests that platelets of AD patients show changes due to upregulation of cortical A2A receptors. In the observations made in postmortem AD patients, A2A expression was found to be higher in AD [295]. The correlation of platelet exchange and A2AR exchange indicates that A2AR may be a new drug target.

A1A receptors may accumulate in neurodegenerative structures in AD, where A1A receptor may mediate both protein processing and phosphorylation and translocation of tau [303]. In fact, upregulation of adenosine receptors in the frontal cortex in AD has been observed, particularly these A1 and A2 receptors [304]. These findings revealed the significance of A1 and A2A receptor regulation with signaling pathways in AD.

Caffeine can block all four subtypes of adenosine receptors. In this context, another study that focused on caffeine’s suppression of Aβ protein precursor (AβPP) internalization and Aβ production via A3 receptor revealed that caffeine partially suppressed the A3 receptor-mediated internalization of the Aβ protein precursor [305]. This effect of caffeine on A3R suppresses LDL cholesterol-induced amyloidogenic processing of AβPP. This suggested that caffeine may exert its protective effects against amyloidogenic processing of the Aβ protein precursor via the A3R. A paper published in 2023 observed the effects of caffeine consumption in a mouse model. Research examining synaptic function, metabolism, and adenosine modulation in different brain regions shows that caffeine consumption does not increase behavior change and synaptic plasticity. Caffeine has been shown to increase synaptic competencies [306]. This property of caffeine may help overcome stimuli that cause brain dysfunction. In 2020, a study found that caffeine from coffee, decaffeinated coffee, and coffee was linked to cognitive performance in older adults. The National Health and Nutrition The Examination Questionnaire (NHANES) used data from 2011 to 2014 [307]. The results of the study reveal the relationship between coffee, caffeinated coffee, and coffee-derived caffeine with cognitive performance. Studying the effects of adenosine and adenosine receptors on AD may contribute to slowing down the neurodegenerative effect of AD (Table 10).

**Table 10:** The role of adenosine in AD based on previous literature

Study type	AD model	Drug and dose	Observation	Remarks	References
Human study	Post-mortem human cortex in AD (*n* = 6) Control group (*n* = 4)	–	Platelet levels change in AD depending on cortical A2AR	The correlation of platelet level change and A2AR level change indicates that A2AR may be a new drug target	[295]
	Post-mortem human cortex in AD (*n* = 31)	–	A1R and A2R have been observed in the post-mortem human cortex	A1R and A2AR regulation in AD may be associated with neuronal signalling pathways	[304]
	Healthy subjects (*n* = 2513)	–	Caffeine intake is associated with cognitive performance	Coffee, caffeinated coffee, and caffeine from coffee may contribute to cognitive performance	[307]
In vitro	Primary cultures of cerebellar granule cells in rats	Caffeine (0.2–25 mm)	A2AR blockade provided neuroprotection in cases of neurotoxicity	A2AR may interact with caffeine in AD	[301]
	Primary cultures of astrocytes from the cerebral cortex of post-natal Wistar rats	SCH 58,261, (50 nM)	The relationship between A2AR and Cx43 regulates the effects of Aβ	The A2AR, Cx43 association can make A2AR activity continuous in AD	[297]
	Primary cultures of neurons and microglia (resting and activated) from control and the APPSw, Ind transgenic mice	–	A2AR antagonists affect NMDA glutamate receptor functions	A2AR antagonists can provide neuroprotection in AD	[302]
	Human neuroblastoma SH-SY5Y cells were cultured in DMEM	–	The effects of A1AR accumulation in AD on protein processing, phosphorylation, and translocation have been studied	Adenosine may be a target for AD	[303]
	Primary cultures of rat cerebral cortical neurons Cultures of human neuroblastoma cells	MRS1334 (100 nM)	Caffeine can suppress A3R-mediated internalization of the Aβ protein precursor	Caffeine can inhibit amyloidogenic processing of the Aβ protein precursor via the A3R	[305],
In vitro, in vivo	Animal model of 3xTg mouse in AD	SCH58261 (0.1 mg/kg per day)	A2AR antagonists have therapeutic-like abilities	The therapeutic-like ability may help treating synaptic disorders and memory dysfunctions in early AD	[293]
	Animal model of APP/PS1 mouse in AD	–	A2AR abolish long-term potentiation at synapses	The therapeutic effects of A2AR antagonists in early AD need to be studied	[299]
In vivo	Animal model of APPswe/PS1dE9 mouse in AD	MSX-3 (0.3 g/L)	A2AR antagonist has been shown to reduce amyloid pathology in AD	A2AR blockade may have therapeutic value in AD	[298]
	Aβ-amyloid (Aβ1-42)-based model of early AD	AOPCP (100 μM), SCH58261 (50 nM)	Overactivity of A2AR is effective in synaptic and memory dysfunction in AD	CD73 activity can be targeted to provide A2AR regulation in AD treatment	[296]
	Male C57bl\6j mice	Caffeinated water (0.3 g/L)	Caffeine consumption affects synaptic function, metabolism, and adenosine modulation	Caffeine’s positive effect on metabolic competence of synapses may help overcome brain dysfunctions	[306]
	Animal model of Aβ-induced cognitive impairment in mice	Caffeine (1 mg/ml), Aβ (1 mg/ml)	Caffeine and A2AR antagonists were effective in preventing cognitive deficits	Caffeine and A2AR antagonists may protect against Aβ-induced amnesia in AD	[300]

*3xTg* triple transgenic model; *Aβ* amyloid β; *A1AR* A1A receptor; *A2AR* A2A receptor; *A3R* A3 receptor; *AD* Alzheimer’s disease; *AOPCP* α,β-methylene ADP; *Cx43* Connexin 43; *DMEM* Dulbecco’s modified Eagle’s medium; *LDL* low-density lipoprotein; *MSX-3*, (E)-phosphoric acid mono-[3-[8-[2-(3-methoxyphenyl)vinyl]-7-methyl-2,6-dioxo-1-prop-2-ynyl-1,2,6,7-tetrahydropurin-3-yl] propyl] ester disodium salt; *NMDA* N-methyl-d-aspartate; *SCH58261* selective A2AR antagonist 5-amino-7-(2-phenylethyl)-2-(2-furyl)-pyrazolo[4,3-e]-1,2,4-triazolo[1,5-c]pyrimidine; *MRS1334* A3R antagonist, 1,4-dihydro-2-methyl-6-phenyl-4-(phenylethynyl)-3,5-pyridinedicarboxylic acid 3-ethyl-5-[(3-nitrophenyl)methyl] ester

## Current Therapeutic Strategy for AD

AD is a complex, multifactorial disease for which the mechanisms still remain to be fully elucidated. The lack of success in a single drug target approach is undeniable that the paradigm of AD drug design needs to be altered [308]. As new insights into AD pathogenesis and progression are gained, developing or repurposing drugs with the capacity to target different aspects of the disease pathogenesis are hence a promising avenue for AD therapies [308]. Over the past two decades, there were only symptomatic treatment available for AD like cholinergic inhibitors and NMDA antagonists, which only relieved the symptoms of the disease without modifying the underlying pathophysiology [309]. Recently, there was a paradigm shift towards development of disease modifying therapies (DMTs) that have been able to target the underlying pathophysiology of the disease and prevent the disease progression at an early stage, with the aim of obtaining an enduring clinical benefit in improving the life for Alzheimer’s disease patients [310]. Several DMTs have been developed and entered the clinical trials; two such drugs, Aducanumab and Lecanemab, have since been approved [311]. Clinical trials for AD target a robust array of biological processes particularly amyloid therapies have shown progress with the approval of the both Aducanumab and Lecanemab. Both FDA-approved drugs have a similar mechanism of action on aggregated amyloid beta, but each drug targets a different form of aggregated amyloid beta and also possess different binding strength towards it, where Lecanemab was shown to have a stronger binding to small and large soluble amyloid beta protofibrils [312]. Protofibrils of amyloid beta have been deemed the main drivers of AD where they result in disruption of membrane integrity and cause synaptic toxicity, and therefore drugs like Aducanumab and Lecanemab that target the protofibrils are deemed disease modifying [313]. However, other amyloid approaches as well as treatments for tau abnormalities, inflammation, and synaptic dysfunction should also be explored in the AD drug development pipeline. As dementia and more severe symptoms occur during the course of AD, other molecular pathways may also be involved in AD progression, thus requiring the use of additional drugs. Thus, there is a need to look at the possible hidden mechanisms and multidrug therapy for AD treatment that uses neurotransmission/neurotransmitter-based therapeutics in combination with the DMTs.

## Potential Limitations Associated with Neurotransmitter-based Treatments

While neurotransmitter-based therapeutics may provide a new therapeutic avenue for AD in the future upon further validation, some of its limitations should be addressed prior to determining its efficacy for AD treatment. One of the major limitations will be that neurotransmitter-based treatments would not be deemed DMTs as they only provide symptomatic relieve by modulating and correcting the levels of neurotransmitter dysfunction in the brain of AD. Moreover, neurotransmitter imbalance is just one of the pathological pathways of AD, which is heterogeneous in nature. Nevertheless, combination therapy with DMTs will not only provide immediate symptomatic relief, which is an advantage over DMTs that may take time to elicit its effects, but reverse or halt the progression of the condition. Besides that, clinical trials, particularly in understanding the long-term effects of neurotransmitter-based treatments, have not been extensive and therefore there is still much research to be done on their potential as therapeutics for AD.

## Conclusion

Neurotransmitters are extremely vital for the survival and preservation of the neuron’s physiological functions. Once the neurotransmitter system has been compromised, neurons are unable to perform their usual physiological tasks, which in turn affects the synaptic plasticity and cognitive functioning. In AD, pathological changes in abnormal neurotransmitter activity or metabolism may occur, which may include the loss of cholinergic neurons, dysfunction of glutamatergic neurons, decrease in GABA levels, loss of monoaminergic neurons, and decrease in monoamine levels, among others. In this review, the role of common but crucial neurotransmitters derived from amino acids, monoamines, gas, purines, and ethanamide and interactions with their respective receptors and transporters were elucidated in the context of AD. In the original articles used, the articles published in recent years were emphasized. In addition, articles using electrophysiological techniques and articles using the new AD model AppNL-G-F mouse model are included. Since neurotransmitters play a key role in the CNS and are widely distributed within the CNS, comprehending the production, transport, and metabolism of neurotransmitter aids in the development of new therapeutic targets for AD. Nevertheless, more research may still be needed on understanding the role of neurotransmitters in AD. Future studies on AD and neurotransmitter may lead to novel treatments and perspectives on AD where multidrug therapy could be a key prospect for AD treatment.

## Data Availability

Not applicable. No data was generated.

## Abbreviations

α7nAChR: α7 Subtype nicotinic acetylcholine receptors
3xTg: Triple transgenic model
5-HT: Serotonin
Aβ: Amyloid β
Aβo: Aβ oligomers
A1AR: A1A receptor
A2AR: A2A receptor
A3R: A3 receptor
ACh: Acetylcholine
AChE/ChEI: Acetylcholinesterase/cholinesterase
AD: Alzheimer’s disease
ADHD: Attention-deficit/hyperactivity disorder
AMPA: α-Amino-3-hydroxy-5-methyl-4-isoxazolepropionic acid
AMPAR: Alpha-amino-3-hydroxy-5-methyl-4-isoxazolepropionic acid receptor
AOPCP: α,β-Methylene ADP
APOE: Apolipoprotein E
APOE*4: Apolipoprotein E*4
APP: Amyloid precursor protein
AR: Adrenergic receptors
Avn-C: Avenanthramide-C
bAR: Beta adrenergic receptor
BDNF: Brain-derived neurotrophic factor
BRET: Bioluminescence resonance energy transfer
BuChE: Butyrylcholinesterase
cAMP: Cyclic adenosine monophosphate
CAPON: C-Terminal PDZ *Ligand* of Neuronal Nitric Oxide Synthase Protein
CD73: *Ecto-5′-nucleotidase*
CDNF: Cerebral dopamine neurotrophic factor
Cef: Ceftriaxone
cGMP: Cyclic guanosine monophosphate
CHT: Choline transporter
CNS: Central nervous system
Compound 23: 2-(5-(Azepan-1-yl) pentyloxy)-9H-xanthen-9-one
CREB: cAMP response element binding
CSD: Chronic sleep deprivation
CSF: Cerebrospinal fluid
CSTC: Cortico-striato-thalamo-cortical
CT: Computer tomography
Cx43: Connexin 43
D1R: Dopamine 1 receptor
D2R: Dopamine 2 receptor
DA: Dopamine
DG: Dentate gyrus
DMEM: Dulbecco’s modified Eagle’s medium
DMSO: Dimethyl sulfoxide
DMT: Disease-modifying therapies
EAAT: Excitatory amino acid transporters
EEG: Electroencephalogram
eNOS: Endothelial nitric oxide synthase
ER: Endoplasmic reticulum
ERK: Extracellular regulated kinase
ET: Extratelencephalic projection
FAB: *Ferrous amyloid buthionine*
GABA: Gamma-aminobutyric acid
GABAaRs: GABAa receptors
GAT: GABA transportes
GBX: Gaboxadol
GIRK: G protein inwardly rectifying K^+^
GSNO: S-Nitrosoglutathione
GTP: Guanosine triphosphate
GluN1: IGluR sub-unit 1
GWAS: Genome-wide association studies
H1R: H1 receptor
HC: Healthy controls
hMAO: Human MAO
Hrh1 Cko: H1 receptor gene conditional knockout
huAPP: Human β-amyloid precursor protein
IC50: Acetylcholinesterase
ICV: Intracerebroventricular
iGluRs: Ionotropic glutamate receptors
i.p: Intrapertoneally
ISIs: Interspike intervals
KAR: Kainat Receptors
KCC2: K + /Cl– cotransporter
LA: L-Arginine
LADDER: Moderate Alzheimer’s disease
LC: Locus coeruleus
LDL: Low-density lipoprotein
LEC: Lateral entorhinal cortex
L-NAME: N(G)-Nitro-l-arginine methyl ester
L-NMMA: A nitric oxide synthase inhibitor
LPS: Lipopolysaccharide
LSD: Lysergic acid diethylamide
L-SPD: L-Stepholidine
LTP: Long-term synaptic plasticity
mAChR: Muscarinic acetylcholine receptors
MAO: Monoamine oxidase
MCI: Mild cognitive impairment
mEPSC: Spontaneous miniature excitatory synaptic current
mGluRs: Metabotropic glutamate receptors
mIPSC: Miniature inhibitory postsynaptic currents
MK801: Dizocilpine
mmPLS: Partial least squares
MSX-3: (E)-Phosphoric acid mono-[3-[8-[2-(3-methoxyphenyl)vinyl]-7-methyl-2,6-dioxo-1-prop-2-ynyl-1,2,6,7-tetrahydropurin-3-yl] propyl] ester disodium salt
MRS1334: A3R antagonist, 1,4-dihydro-2-methyl-6-phenyl-4-(phenylethynyl)-3,5-pyridinedicarboxylic acid 3-ethyl-5-[(3-nitrophenyl)methyl] ester
MTDL: Multi-target-directed ligands
MTG: Middle temporal gyrus
NAc: Nucleus accumbens
nAChR: Nicotinic acetylcholine receptors
NE: Norepinephrine
NET: Norepinephrine transporter
NFT: Neurofibrillary tangles
NHANES: National Health and Nutrition Examination Survey
NMDA: N-Methyl-d-aspartate
NMDAR: N-Methyl-d-aspartate receptor
nNOS: Neuronal nitric oxide synthase
NO: Nitric oxide
NOR: Novel object recognition
NOS: Nitric oxide synthase
NSC: Neural stem cells
ORM: Object recognition memory
PAP: Passive avoidance paradigm
PD: Parkinson’s disease
PEA-OXA: 2-Pentadecyl-2-oxazoline
PEI: Polyethylenimine
PET: Positron emission tomography
PFC: Prefrontal cortex
PH-TMN: Posterior hypothalamus-tuberomamillar nucleus
PKA: Protein kinase A
PKC: Protein kinase
PLC: Placebo
PRMT4: Protein arginine N-methyltransferase-4
PrPC-mGluR5: Cellular prion protein-metabotropic glutamate 5 receptor
PS1-De9: Presenilin 1
PTZ: Pentylenetetrazole
RNS: Reactive nitrogen species
RTG: Dopamine antagonist rotigotine
RVT: Rivastigmine
SCH58261: Selective A2AR antagonist 5-amino-7-(2-phenylethyl)-2-(2-furyl)-pyrazolo[4,3-e]-1,2,4-triazolo[1,5-c]pyrimidine
SCN: Suprachiasmatic nucleus
SDB: Sleep-disordered breathing
SERT: Serotonin transporter
sGC: Soluble guanylate cyclase
SNpc: Nigra pars compacta
SSRI: Elective serotonin reuptake inhibitors
TMN: Tuberomammillary nucleus
TRKB: Tropomyosin-related kinase receptor type B
VAChT: Vesicular acetylcholine transporter
VTA: Ventral tegmental area
WT: Wild type
